# Adapt-Ed: co-designing adaptations to a whole school intervention to improve the uptake and impact of food provision in special schools – scoping research for a future trial

**DOI:** 10.3310/nihropenres.13897.2

**Published:** 2026-08-01

**Authors:** Rebecca O'Connell, Amanda Ludlow, Angus Holford, Laura Hamilton, Louca-Mai Brady, Lauren Denyer, Andy Feltham, David Wellsted

**Affiliations:** 1 University of Hertfordshire, Hatfield, England, UK; 2 University of Essex, Colchester, England, UK

**Keywords:** School food, whole school food environments, special educational needs and disabilities, children and young people, health inequalities, co-production, mixed methods

## Abstract

**Background:**

Children and young people (CYP) with Special Educational Needs and/or Disabilities (SEND) are more likely to grow up in poverty and be entitled to free school meals than other children. However, they might not eat well at school because their needs aren’t always accommodated. There is a need to understand school food provision for children with SEND and how the whole school food environment (WSFE) can be improved to address health inequalities.

**Methods:**

We conducted scoping research and involved public and professional stakeholders in co-producing adaptations to a whole school food approach called Healthy Zones (HZ). The aim was to inform a future proposal to implement and evaluate the Adapt-ed HZ intervention in special schools. The four work packages (WP) (June to November 2024) were: WP1: establish stakeholder groups to co-produce a logic model and HZ adaptations; WP2: scoping reviews of food interventions for CYP with SEND; WP3: analysis of publicly available administrative data and online survey; WP4: ethnographic case studies of three special schools.

**Results:**

Working with CYP with SEND, parents, carers and professionals in schools and policy roles, Adapt-Ed has provided a wealth of information to tailor and refine the HZ approach for special schools. It has included extensive public involvement and scoping research to inform future research. The work has led to a draft logic model and outline research plan which have been co-produced with public and professional stakeholders throughout.

**Conclusion:**

Through this project we have co-produced HZ adaptations and a draft logic model and learned there is a need and demand for Adapt-Ed HZ to enhance WSFEs in special schools and promote children’s health and wellbeing. Subject to a feasibility study to refine the research protocol and test data collection methods, a trial is recommended to implement and evaluate the effect of Adapt-Ed HZ on the health and wellbeing of children with SEND.

## Plain Language Summary

Why are we doing this project?

Eating nutritious food at school can create more equality in the diets and lives of children from lower and higher income families. However, children with special educational needs and/or disabilities (SEND) might not be able to eat well at school because their needs aren’t always accommodated.

Schools can have an influence on children’s eating habits, including education and activities outside school. Making learning about and eating nutritious food part of all aspects of the school day is known as a ‘whole school approach’. This approach can increase the number of children who eat school dinners and improve their health and wellbeing. We wanted to explore how a ‘whole school approach’ called Healthy Zones might work in special schools.

What have we done so far?

We involved children and young people, their caregivers, and people who work in or for schools in our plans. This helped us to create an idea of what the approach needs to grow and have good outcomes.

We reviewed past research to identify ways to measure nutritious eating for children with SEND. This helped us discover that there are not enough ways to measure nutritious eating for children with SEND.

We explored existing information on free school meals and found out that children with SEND are more likely to be eligible for free school meals, but less likely to have one in some schools.

We worked with three special schools in the East of England to find out how taking part in future research would work for them. This helped us identify the opportunities and practicalities of trying out a whole school approach in future research.

What is next?

We will use what we’ve learned to apply for a new project to test our Adapt-Ed Healthy Zones in special schools.

## Background

Internationally, schools are recognised as determinants of children’s health and wellbeing, both directly, as food providers, and indirectly, through delivering health and food education ( Currie *et al*., 2012; Oostindjer *et al*., 2017). Consistent with the World Health Organisation’s Health Promoting Schools framework ( Langford *et al*., 2017; WHO, 1997, 1998), the UK government recognises that schools are well-placed to address inequalities in dietary health, mandating that state funded schools follow School Food Standards (SFS) ( DfE, 2014, 2023) and recommending they create and implement ‘whole school food’ policies ( HM Government, 2022:205). Whilst there is currently no routine monitoring of SFS, or other aspects or impacts of school food provision and take up in England ^1^ ( FSA, 2023), a growing body of research suggests nutritious school meals can meet children’s ‘right to food’ and mitigate the effects of poverty and inequality on the diets and lives of children and low-income families ( O’Connell *et al*., 2022), including improved family finances ( Holford & Rabe, 2020). However, there is less evidence about variation in access to, or impacts of, school food provision by intersecting health and social inequalities among children and young people, including those related to disability.

In the UK, children with special educational needs and/or disabilities (SEND), as defined by the Children and Families Act 2014, are more likely to grow up in poverty, and almost twice as likely to be entitled to means-tested free school meals (FSM), than other children ( DfE, 2023; DWP, 2023; JRF, 2023). Many children with SEND have eating difficulties, fall outside their suggested healthy body mass index (BMI) ( Smith & Ludlow, 2022), and are at greater risk of being overweight, underweight and malnourished, particularly those with more restrictive and rigid patterns of eating ( Smith *et al*., 2019). Children with SEND and their families are therefore especially likely to benefit from nutritious school food provision ( Brannen *et al*., 2024). However, the charity Contact (2023) found a third (33%) of 1,500 surveyed families with a disabled child eligible for FSM could not access their FSM for various reasons, with the greatest proportion due to “sensory and dietary needs”.

In England, around 1.7 million school pupils (18% of all pupils) are identified as having Special Educational Needs (SEN) ( DfE, 2024b; Long & Roberts, 2025:6). Of these, around 1.2 million receive SEN Support, and around 0.4 million have Education, Health and Care (EHC) Plans (Ibid.). SEN support is given to children and young people in their pre-school, school, or college; EHC Plans are for children and young people aged up to 25 who need more support. The number of pupils with EHC Plans and SEN support has grown exponentially in the last ten years, with the largest rises related to autistic spectrum disorders ^2^ ( NAO, 2024:32). Education for children with SEND takes place across mainstream and specialist settings, with variation in terms of the types and complexity of needs provided for. Whilst *most* children with EHC Plans are taught in mainstream schools, *all* children in special schools have EHC Plans. This means that special schools have the most experience in food provision for children with SEND and are best-placed to lead the way in making improvements. However, as recognised by recent government spending commitments ( Gov.UK, 2024), the specialist education sector also faces enormous challenges including rising demand for places (Education Committee, 2024; Public Accounts Committee, 2025) with high food costs and staff turnover particularly relevant for special schools given the range of needs and high staffing ratios ( Bremner *et al.*, 2024; SFM, 2024; Whittaker, 2023).

There is some evidence that systemic, Whole School Food Environment (WSFE) approaches that involve the whole school community, and make food part of everyday school life, can improve the take up of school meals and impact on health and other outcomes ( Brennan *et al*., 2021; Forbes *et al*., 2025; Moore *et al*., 2023). However, research needed to support and evaluate improvements to WSFE has almost exclusively been carried out in mainstream schools and excluded children and young people with SEND; there is little systematic evidence about school food context, take up of meals, the impacts of food in special schools, or how these might be improved. Funded by a National Institute of Health and Care Research (NIHR) Application Development Award (ADA), *Adapt-Ed* (June-Dec 24), set out to begin to address this gap.

## Aim and objectives

The aim of Adapt-Ed was to carry out scoping research, including workshops with children and young people with SEND, parents and carers, special school staff and other stakeholders, to co-produce adaptations to an existing WSFE programme, Healthy Zones (HZ), and inform a future proposal to NIHR.

HZ is a whole school approach that was devised and is delivered by the charity School Food Matters (SFM), a project partner. In the existing HZ model schools begin with a ‘school food audit’, the findings of which are used to create an action plan and school food policy. Dedicated HZ Project Officers (POs) support the school to progress their action plans, embed positive changes, and engage the whole school community in the work. In the future study we propose to implement the Adapt-Ed HZ programme in special schools in the East of England (EoE) and evaluate its effectiveness in improving children’s dietary intake and other outcomes and impacts at the level of the child, family and school.

The objectives [OBJ] of the ADA were to:

1. Establish public involvement and professional stakeholder groups to co-produce a logic model, co-design adaptations to HZ, and inform the future plans and proposal (WP1)
2. Review existing interventions and identify appropriate dietary outcomes and measurement methods for this population (WP2).
3. Identify relevant aspects of school context in order to provide a sampling frame for the future evaluative and intervention research in the East of England and examine whether a Stepped Wedge trial is an appropriate design (WP3).
4. Explore special school contexts, including what data seem relevant and feasible to collect from schools, pupils and families to evaluate intervention effectiveness (WP4).
5. Develop a draft proposal to evaluate the effectiveness of co-designed adaptations to an intervention to improve the uptake and impact of food provision in special schools.

## Methods

### Patient and Public Involvement

Public involvement and engagement informed the preparation of the ADA proposal, and the involvement of children and young people with SEND, parents and carers was embedded throughout the project, underpinned by the UK Standards for Public Involvement in Research ( HRA, 2020). Public involvement was the focus of Work Package (WP) 1, that was co-led by AF, as public co-applicant with relevant lived experience. Further details regarding when and how the public were involved in the project are discussed in the sections on WP1 below in Methods; the outcomes from, and impact of, this involvement are covered in the Results and Discussion.

The study took a mixed methods approach, consisting of four parallel Work Packages (WP):

***WP1 Public and stakeholder involvement (to address OBJ1).*** The ADAPT guidance on adapting interventions to new contexts recommends collaboration with stakeholders from the earliest stages ( Moore *et al*., 2021). HZ was devised and is delivered by the charity School Food Matters (SFM), a partner on the grant. In consultation with SFM we identified stakeholders most affected by this research and with power to influence school food in special schools as within two main groups: (1) ‘public contributors’ including: (a) children and young people with SEND and (b) their parents and carers; and (2) ‘professional stakeholders’ including those working in (a) schools (teachers, school nurses, governors/trustees, catering and support staff ) and (b) education and health policy and strategy (local authority policymakers, commissioners, voluntary/community sector organisations, catering providers) ^3^.

Recruitment to stakeholder groups was carried out via a range of methods including general articles and announcements online (e.g. an article on the NIHR ARC East of England website), notices in newsletters (e.g. SFM newsletter, local authority mailing lists), emails to network convenors and existing contacts, and personal email invitations to individuals. We recognised the need to access children and young people with SEND via gatekeepers. Whilst we did not aim for ‘representativeness’ (as our purpose was not to generalise) we did want to achieve diversity of experience and expertise. We therefore collected optional demographic data, and took an iterative approach, targeting public and professional members who contributors told us were currently ‘missing’. We aimed for around six people in each group, which is ideal for focus-group type workshops ( Bryman, 2004). We aimed to ensure any access needs were met, and included time and space during workshops to get to know each other, build trust, learn and share ideas. However, we set out to prepare detailed plans in collaboration with those involved. In particular, we recognised that person-centred involvement with children and young people, is most effective when developed with young people, rather than expecting them to fit into a model pre-determined by adults ( Cuevas-Parra, 2020).

The gatekeepers (services and parents and carers) and children and young people we spoke to early on made clear that our initial plans to organise a project-specific young people’s group would not work for them in terms of access, inclusion or availability, so plans for involvement were revised in collaboration with those we wished to involve, and organisations working with them. We aimed to make participation as flexible and inclusive as possible, with ‘pockets of participation’ depending on young people’s interests, availability, and personal circumstances ( Franks, 2011). In practice this meant we contacted a variety of organisations working with children and young people with SEND with information about the project and, during an initial meeting, found out more about the group and how they could be involved in ways that worked for them.

1. Public contributors

a. Parents and carers (n = 6)

We formed a group of six parents and carers of children with SEND, who met with us online three times, for around 90 minutes at each workshop, between July and December. The group included a mix of in terms of gender, ethnicity and child’s needs regarding food and eating. However, most were mothers, White British, with ASD/autism related eating difficulties most common among their children. The latter attended a variety of educational provision, including mainstream school, special schools, and Education Otherwise Than at School (EOTAS). In addition, we met with 17 parents in two special schools as part of WP4. Of those parents who shared demographic information, most identified as being on a low income (defined ‘subjectively’ as below what they needed ( Gordon, 2006:51) and/or having a child eligible for FSM) (see Table 1 for a breakdown of meetings and attendance).

**Table 1.** Public contributors and professional stakeholder involvement Work Package (WP)1 and WP4.

WP1 Involvement and Group Meetings ^**1**^				WP4 School Visits ^**2**^
***Public Contributors***				
a) Parents and Carers (n = 6)	**Meeting 1** (September): n = 5 attended (n = 1 sent comments by email)	**Meeting 2** (November): n = 4 attended	**Meeting 3** (December): n = 3 attended	*Parents and Carers (n = 17)* **School A:** Group discussion (n = 10)
				**School B:** Phone call (n = 1)
				**School C:** Existing coffee morning (n = 6)
b) Children and Young People with SEND (n = 42)	**SEND Youth Forum A** (September): In-person workshop with 14 children and young people aged 11–25 years.			
	**SEND Youth Forum B** (October): In-person workshop with 10 children and young people aged 11–25 years.			
	**SEND Youth Forum C** (November): Online workshop with 10 children and young people aged 13–21 years.			
	**Special School’s Youth Council** (October): In-person workshop with 7 children and young people aged 4–18 years.			
	**Individual Meeting** (December): with 1 young person aged 12 years.			
***Professional Stakeholders***				
a) People working in schools (n = 7)	**Meeting 1** (July): n = 3 attended	**Meeting 2** (October): n = 5 attended	**Meeting 3** (December): n = 3 attended	
b) People working with schools and policy (n = 17)	**Meeting 1** (September): n = 11 attended	**Meeting 2** (October): n = 8 attended	**Meeting 3** (December): n = 8 attended	

^1^ Purpose of each meeting were as follows: **Meeting 1** introduction to the project and group (90 minutes); **Meeting 2** developing the sunflower model (90 minutes); and **Meeting 3** summarising the model and next steps (90 minutes).

^2^ Engagement activities carried out during WP4 ethnographic visits to three case-study special schools.

b. Children and young people (n = 42)

Between September–November, we met with 41 children and young people (aged 4–25 years old), through attending three youth groups for children and young people with SEND, a session with a School Council within a special school, and a 1:1 meeting, using a mix of modes (in-person and online), ranging from 30 to 90 minutes. Our approach was underpinned by the Lundy (2014) model of participation which is focussed around four core concepts (space, voice, audience and influence) all of which need to be in place to meet young people’s rights to be heard in both personal and public decision making. By ‘voice’ we mean communication, in whichever form works best for the child or young person. Broad topics and questions were discussed with group facilitators beforehand and sent ahead of the meeting. During the sessions we used a variety of methods to gather insights from CYP with SEND, including question sheets with widgits (created using SymWriter 2 software), communication via communication aids, or augmentative and alternative communication (AAC) devices, small group discussions and creative methods (e.g., colouring sheets of food pictures and coloured pens to encourage conversations about food). We also ensured that adults who knew the CYP best were present during these conversations to help facilitate conversation and create a safe space, given that we had only limited time to form relationships with group members ( Fields et al., 2025). Due to the project timeline, and capacity and resources of each group, we were only able to meet with each group on one occasion during the funded period ( Table 1).

2. Professional Stakeholders

a. People working in schools (n = 7)

This group included three school Chefs/Cooks, a Head of Food Studies, a Deputy Head Teacher, a Teaching Assistant (all in special schools), and a Public Health Officer working with schools.

b. People working in policy and with schools (n = 17)

This group included representatives of Trades Unions, parent/carer networks, catering companies (including a Local Authority Trading Company, LATCo ^4^), charities for disabled children and their families, local authority Public Health Officers, the Department for Education, and the Department of Health and Social Care.

We met with each professional stakeholder group separately, twice, between July–November, then together, with members of the parent/carer group, at a joint meeting in December. Each meeting lasted around 90 minutes ( Table 1).

***Wider stakeholder engagement (approx. n = 41)***

Alongside our ‘core’ stakeholder group meetings (above) we held ‘ad hoc’ 1:1/small group meetings with professionals and organisations identified via our scoping research and networking. This included nine individual meetings with approximately 41 people in total, including the Special Educational Consortium, The Royal College of Speech and Language Therapists, and people in Public Health and Education related roles in various local authorities.

In line with our ethical approval, online stakeholder engagement was automatically recorded, with contributors’ informed consent, and/or notes were taken. Automatic transcriptions of core stakeholder workshops were corrected against audio recordings by a professional transcriber. Discussions with children and young people, and most wider stakeholder engagement, was recorded in fieldnotes and written up immediately afterwards. At least one member of the research team completed a Patient and Public Involvement (PPI) log ( PIHWE, 2023) after each meeting. For all core stakeholder workshops, we asked for members’ feedback about what worked well (or not) and sent summaries via email of what we thought we had learned from our discussions. A simplified ‘Framework’ approach ( Gale *et al*., 2013) was employed to organise and make sense of information gathered (transcripts and notes of discussions), charting this against predefined topics/questions whilst leaving room for unanticipated discoveries.

***Growing Healthy Zones***

**Figure 1.**
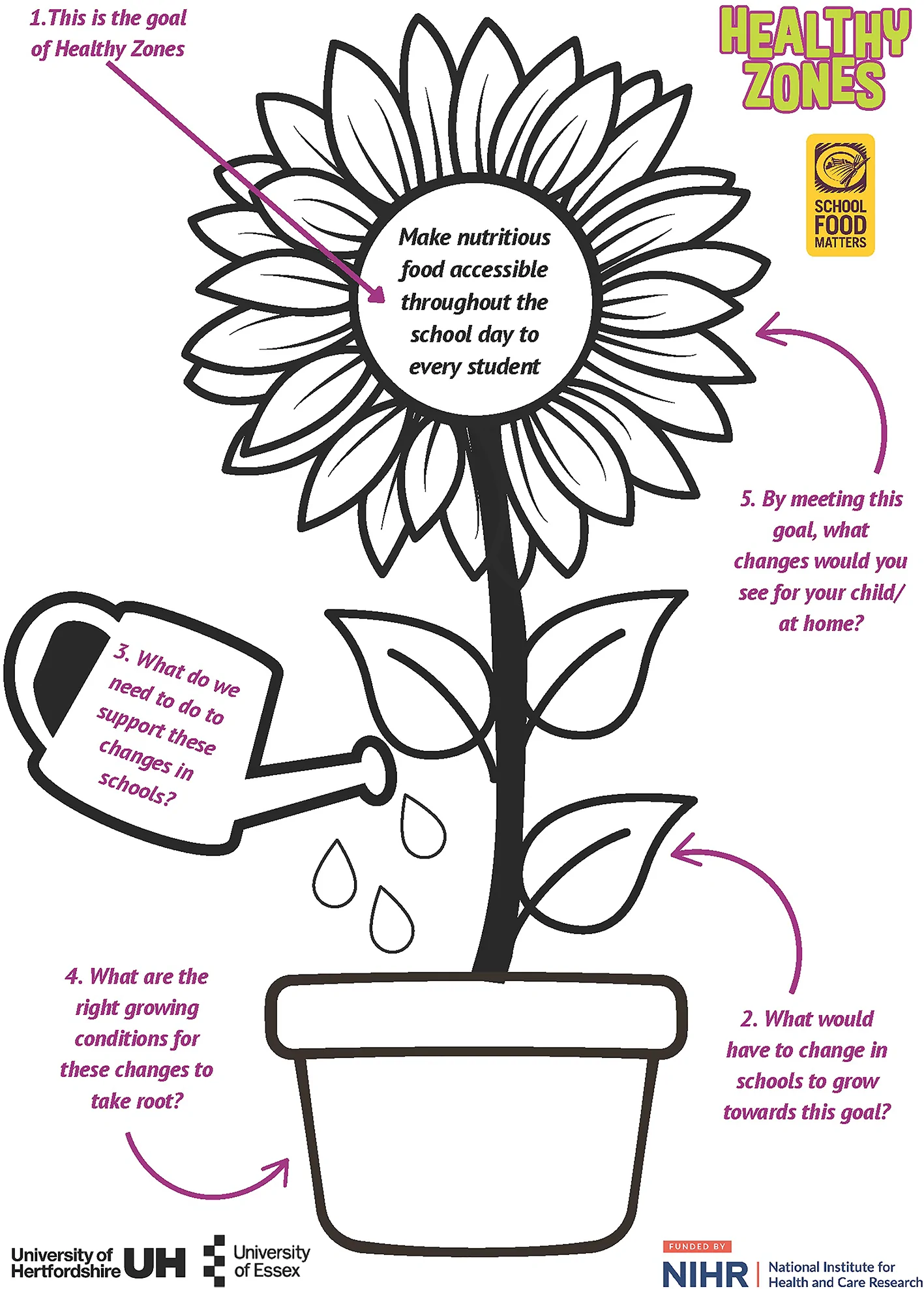
‘Growing Adapt-Ed Healthy Zones’ sunflower metaphor used to co-develop the final collaborative logic model. Each part of the sunflower represented a specific element for the final collaborative logic model, as follows:
1.Centre of Sunflower: the overall goal or aim of the HZ programme.2.Leaves: the changes that need to occur within schools to achieve the intended goal.3.Water and fertilizer: the activities and/or support required to make these changes.4.Pot and soil: the inputs and resources required to help schools make these changes.5.Sunflower petals: the short-, medium- and long-term outcomes and/or impact of the programme (i.e. benefits arising from the activities).

Central to our approach to working with stakeholders was the co-production of an Adapt-ed HZ logic model. Logic models and theories of change are ways of identifying the inputs and activities needed for an intervention to achieve the desired results and are commonly used to plan and design evaluative research. Collaborative logic modelling is one way that public contributors and professional stakeholders can be involved with the design of research, policies and services ( Harris *et al*., 2018). Logic models and theories of change can be represented through metaphors, as these are widespread in everyday language and allow us to understand abstract thoughts and feelings that cannot be directly seen, heard, touched, smelled, or tasted ( Lakoff & Johnson, 1980, 1999). Commonly used metaphors for logic models in consultations with public contributors are ‘roadmaps’ and ‘trees’ (e.g. Breen, 2023; CLAPS, 2024). In this project, we considered a variety of examples, including ‘baking a cake’; we decided to use a sunflower to present the conditions, steps and outcomes of ‘growing Healthy Zones’, that was suggested by our collaborators at SFM, as it seemed clear and straightforward to translate into a traditional logic model format ( Figure 1). We only used this with adult groups, however, as when we discussed with young people with SEND, they reflected that sunflower metaphor made the discussion more confusing, not less complicated. Removing the sunflower metaphor and using a simple explanation of key programme elements when discussing it with young people with SEND made the research more accessible to them.

The process of co-producing the sunflower logic model had a number of stages. A separate model was initially co-produced with each adult group, at the second in a series of three workshops with each. (We only met with each group of children once during this stage of the project, and as noted did not introduce the logic model to our discussions). These separate models were then combined, and at this stage input from discussions with children and young people groups was added, along with any additional input from our wider stakeholder engagement. A key was used to indicate the source of the input, and show where it was contributed by more than one set of core stakeholders. The combined model was shared with representatives of each (adult) group in a joint third meeting, at which it was revised and refined, and consensus was reached regarding its completeness.

***WP 2 Scoping Reviews of relevant interventions, outcomes and dietary assessment methods (to address OBJ2).*** Our initial reviews of the literature identified little existing research on school meal provision or experiences for children with SEND and no comprehensive reviews of evidence on food interventions with SEND children, appropriate dietary assessment methods, or outcome measures for this group. We set out to carry out scoping reviews ( Grant & Booth, 2009; Munn *et al*., 2018) since these are considered the most appropriate review method to understand the scope and nature of a body of literature that spans disciplines and designs and has not been comprehensively reviewed ( Brock, 2023; Peters *et al*., 2015).

We conducted two scoping reviews, incorporating academic literature to inform the future evaluative research, specifically study design, including identification of interventions and appropriate instruments and outcomes related to food behaviours and dietary intake for children with SEND ^5^. Protocols for both scoping reviews were designed with reference to the Joanna Briggs Institute (JBI) methodological guidance for scoping reviews and JBI Manual for Evidence Synthesis ( Aromataris *et al*., 2024; Peters *et al*., 2020), reported in accordance with the Preferred Reporting Items for Systematic Reviews and Meta-Analyses extension for Scoping Reviews (PRISMA-ScR; Tricco *et al*., 2018), and registered with the Open Science Framework ( Denyer *et al*., 2024a, 2024b). Searches were conducted on PubMed, Scopus, EMBASE, Web of Science and PsycINFO databases. Using the software, Rayyan, the references were screened by two researchers independently and any discrepancies resolved via a discussion with a third reviewer. Data were extracted into a table and accompanied by a narrative summary.

**Scoping review 1** ( Denyer *et al*., 2024a) aimed to examined and synthesis the existing literature concerning interventions to improve the diets, eating and mealtime behaviours of children and young people with SEND in any setting. Inclusion and exclusion criteria are set out below using the Participants, Concept, Context (PCC) mnemonic ( Table 2).

**Table 2.** PCC inclusion and exclusion criteria for scoping review 1 (interventions).

Participants	Inclusion: Children and young people with Special Educational Needs and Disabilities as defined by the Children and Families Act (2014) aged between 4–24 years old. There were no restrictions on the sex, ethnicity or socio-economic status of the children with SEND. Exclusion: Children aged under 4 years and over 24 years.
Concept	Inclusion: Interventions aimed at improving the diets (food, food groups, nutrients, dietary quality and dietary diversity), eating and mealtime behaviours of CYP with SEND. Exclusion: Interventions that do not address diets, eating or mealtime behaviours. Clinical in-patient treatments.
Context	Inclusion: The study can be conducted in any setting (e.g. home, school, specialist provision or hospital setting). No restrictions on geographical location. Exclusion: Interventions conducted with groups of less than 5 participants.

**Scoping review 2** ( Denyer *et al*., 2024b) aimed to examine and synthesise the existing literature concerning how diet, eating, and mealtime behaviours are assessed in children and young people with special educational needs and disabilities. Inclusion and exclusion criteria are set out below using the PCC mnemonic ( Table 3).

**Table 3.** PCC inclusion and exclusion criteria for scoping review 2 (outcome measures).

Participants	Inclusion: Children and young people with Special Educational Needs and Disabilities as defined by the Children and Families Act (2014) aged between 4–24 years old. There were no restrictions on the sex, ethnicity or socio-economic status of the children with SEND. Exclusion: Children aged under 4 years and over 24 years.
Concept	Inclusion: Assessment methods, tools and outcome measures used to assess diet (food, food groups, energy and nutrients, dietary quality and dietary diversity) and eating behaviours in children and young people with SEND. In addition to any other outcome measures and tools used within the study. Exclusion: Assessment of other conditions or do not include any dietary, mealtime or eating-related assessment.
Context	Inclusion: The study can be conducted in any setting, e.g., home, school environment or hospital setting. No restrictions on geographical location. Exclusion: None.

***WP3 Typology of Special Schools in the East of England (to address OBJ3).*** In England, there is no current, comprehensive survey, or routinely collected data, on school characteristics, practices and outcomes of school food provision. This initial stage therefore explored what existing administrative data could tell us about the overall picture of school food provision, whether new survey data could fill in any gaps, and if together this information could help inform the future study design and sampling strategy. Specifically, we wanted to know whether we could identify and measure relevant school characteristics in order to stratify them in our future trial design ^6^. We also wanted to look at the typicality (or not) of East of England (EoE) ^7^ schools’ characteristics and outcomes relative to the rest of the country.

A main outcome of interest was take up of school meals. This is a pragmatic proxy for exploring the impact of school meals on pupil outcomes, since the association between take up of school meals and child health outcomes is well-established ( Holford & Rabe, 2020, 2022, 2024). Although there are no publicly available data on the take up of all (paid and unpaid) school meals in England, take up of means-tested and Universal Infant FSM (among those eligible) is routinely collected and publicly available. However, an important limitation is that this indicator is based on data collected on one day of the year – ‘census day’ – for the purposes of allocating core funding, hence it may not be representative of take up generally. We therefore also sought to explore whether we could fill any gaps with an online survey of schools in the EoE region.

To describe special schools in EoE, by relevant characteristics, and explore associations with means-tested and Universal Infant FSM take up among those eligible (hereafter FSM take up), we:

(a) combined routinely collected public data for analysis to create a cross-sectional dataset of (currently open) state-funded special schools, Pupil Referral Units (PRU) with specialist SEN provision and mainstream schools with a SEN Unit, Resourced Provision or Special Classes across England. The combined public data include the January 2024 Department for Education ‘underlying data’: ‘ **Get Information About Schools**’ (GIAS; DfE, 2024a) resource (accessed 14 ^th^ August 2024) and ‘ **Schools, Pupils and Characteristics**’ dataset (SPC; DfE, 2024b). We conducted descriptive analysis of eligibility and take up rates of means-tested FSM (all ages) and of Universal Infant FSM (Reception, Year 1, 2); and Ordinary Least Squares regression to explore the school-level predictors (such as ethnic composition of school intake and urban/rural location) of take up of means-tested FSM among FSM-eligible children (all ages), and of Universal Infant FSM among all infant children. We compare differences between school types within EoE; and within school types between EoE and the rest of England. Since we are interested in the explanatory power of school-level variables, and potential for school-level changes in policy, we give each school equal weight in the regression rather than weighting observations in proportion to the number of (FSM-eligible or infant) children in each school.

(b) designed and conducted an online survey of state-funded special schools and mainstream schools with a SEN Unit, Resourced Provision or Special Classes in EoE (N = 341 eligible) using JISC Online Surveys. WP1 stakeholders and SFM provided input when designing the survey. We aimed to i) cover aspects of school food provision, policy and practice, and support for pupils’ SEND, that are not collected in the routine public data; and ii) establish interest in the future Adapt-Ed intervention and willingness to be recontacted. We identified and contacted local authority and county council contacts with responsibility for SEND and/or schools with information about the survey (including the online link) and asked them to circulate amongst eligible schools. Relevant schools networks in the region were also contacted. Following this, survey invitations were sent directly to head teachers or relevant SENCo and admin staff (if contact details were publicly available) using the JISC automatic distribution tool.

Stakeholders recommended incentives to encourage participation. Those who completed the survey could choose to enter their school into a prize draw to win one of three prizes (a £500 Love2Shop voucher, a consultation with SFM about ‘sensory gardens’ or a £50 National Garden voucher). The relatively small number of responses (n = 33/341; a 9.7% response rate), despite recommended use of incentives, was anticipated ( Sturgis *et al*., 2006). The small number of schools responding limited the kinds of statistical models (using regression methods) that could be used. There is a large number of determinants related to take up of school meals, making any model with small numbers of respondents difficult to interpret.

(c) conducted an ‘audit’ of EoE special schools’ websites (N = 114) to explore what other relevant information could be gleaned from school websites. Specifically, we examined whether the following were available online for each school: (i) a written school food policy; (ii) in-house or external catering provision details (and if external, which provider); and (iii) current lunch menus. These topics were informed by the emerging findings across the various WP, including indicative findings from the online school survey. For pragmatic reasons, only special schools in EoE were included in the audit. Descriptive analysis was then carried out.

***WP 4 Ethnographic case studies of food provision in three schools (to address OBJ4).*** We purposively selected three EoE special schools (in Norfolk, Hertfordshire and Essex ^8^) to provide a contrast of contexts in terms of area deprivation (according to the Index of Multiple Deprivation, IMD), proportion of pupils eligible for FSM (a pragmatic proxy for low-income, ( Ilie *et al*., 2017; Taylor, 2018)) and ethnic mix. We also sought to include schools in different settings in terms of their urban/rural location and type of catering provider.

As shown in Table 4, the three schools selected are all state funded ‘through’ schools catering for children from infants (or in one case, a nursery) up to the age of 19 years. All are mixed in terms of gender, with a greater proportion of males than females (around 3:1 in each case, reflecting the national picture). All three schools provide for children with Autistic Spectrum Disorder (ASD), however, they vary in terms of their other specialisms, and in their location, the proportion of children eligible for FSM, the ethnic mix of the school population, and catering arrangements:

• School A is a medium sized special school (<200 pupils) for children aged 4–19 years, in a coastal area with high levels of deprivation compared to the rest of England (according to IMD 2019 data at the local authority level). In addition to ASD, it provides for children with a range of learning difficulties and impairments. Characteristic of the local demography, the large majority of pupils (>80%) are classified as White British, and the proportion of children eligible for a free school meal (FSM) (>40%) is above average compared to other special schools (East of England average 44.5%). Catering for school meals is provided by the arm’s-length Local Authority catering provider (or Trading Company (LATCo)). Take up of FSM is reported to be 80–100 per cent of eligible pupils ^9^.
• School B is a smaller school (<100 pupils) for children aged 2–19 years in a relatively affluent area compared to the rest of England. This school was selected to provide for a contrast with School A in terms of eligibility for FSM (20–40%, which is below average for special schools) and ethnic diversity, with less than 60 percent of pupils classified as White British. The school also differs from School A in that it specialises in provision for children and young people with more profound and multiple and severe learning difficulties, as well as a range of impairments. It is also contrasting with School A in that catering is provided in-house. Take up of FSM is reported to be under 20 per cent of eligible pupils.
• School C is a larger school (>300 pupils) for children aged 5–19 years, in a rural town location. Whilst the local authority area in which the school is located has lower levels of deprivation compared to the rest of England, the catchment includes some of the most deprived areas in England. In contrast to Schools A and B, this school provides for a much smaller range of learning difficulties. Around half of the children are eligible for a FSM (40–60%), and, as in school A, the large majority of the children (>80%) are White British. As in School B, catering is provided in-house. Take up of FSM is reported to be over 80 per cent of eligible pupils.

**Table 4.** Characteristics of the three Work Package (WP) 4 case-study schools.

	School A	School B	School C
**Location Type**	Rural town/fringe	Urban City/Town	Rural town/fringe
**Local Authority Index of Multiple Deprivation 2019** ^**1**^	Decile 5	Decile 8	Decile 6
**No. of Pupils (% male)** ^**2**^	100–199 (60–70%)	< 100 (60–70%)	300–399 (60–70%)
**No. of Special Educational Needs (SEN) Types Supported**	Nine	Six	Two
**Age Range**	4–19 years	2–19 years	5–19 years
**Ethnicity (% White British (WB))**	80% – 100% WB	40% – 60% WB	80% – 100% WB
**Free School Meal (FSM) Eligible (%)**	40% – 60%	20–40%	40–60%
**FSM Take Up (%)** ^**3**^	80–100%	0–20%	80–100%
**Catering Provider**	Arm’s-length Local Authority Trading Company (LATCo; cook employed by LATCo).	In-house catering provider (cook employed by school)	In-house catering provider (cook employed by school)

Note: Details for each school identified from WP3 analysis of public admin data and website audit. A range for each indicator is provided to maintain schools’ anonymity.

^1^ Higher deciles equate to lower levels of deprivation relative to other local authorities.

^2^ Excluding nursery and sixth form, where applicable.

^3^ Reported take up of FSM for those eligible for FSM.

Our approach, using a mix of qualitative methods including participant observation, is consistent with a broadly ethnographic design, however we did not have the resources in this ADA to fully immerse ourselves in school settings over an extended period of time. Rather our approach was more akin to the early stages of a larger ethnographic study, often referred to as ‘scoping the field’, in which we made short visits to the schools, purposively sampling days, situations and people, to familiarise ourselves with the settings and identify relevant aspects of context for the intervention and future study ( O’Connell, 2016). We visited Schools A and B for two days each and School C for (part of ) one day ^10^ to explore *school policies, provision and practices within these different contexts* and examine the *views of staff and parents* on a) opportunities and challenges for school food provision, b) what improvement would look like, and c) acceptability and feasibility of the future intervention/evaluation. Two researchers visited each school, one of whom (ROC) visited all three schools, with a third researcher (LMB) joining the visit to School A to facilitate the involvement of the School Council in the WP1 (public involvement) discussion. We asked schools for their support for us to hold a parents and carers coffee morning, and to help us recruit parents for this, using a flyer we prepared. We also asked to interview key members of staff and observe mealtimes, snack times, and other food related activities. (Anonymised recruitment materials are provided in supplementary files).

As shown in Table 5, we used a range of methods ( Hammersley & Atkinson, 2019; Spradley, 1979) including participant observation to engage with different target groups (staff, parents and carers, and children and young people) in each school. We prepared and referred to observation and topic guides that directed our gaze and questions, but we were also led by interests and opportunities that arose in each setting. With staff, we conducted formal (semi-structured) interviews (three Headteachers, a Deputy Head, two School Cooks and an Area Catering Manager) and carried out informal (less structured and unrecorded) discussions with others (a Deputy Head, two Educational Psychologists, four Teaching Assistants (TAs), a School Nurse and a School Cook). A Family Support Worker (FSW) helped facilitate our coffee morning in School A and also took part in the discussion. Seventeen staff took part in formal interviews and informal discussions in total. We met with parents and carers in two schools during a bespoke coffee morning (School A, 10 parents and carers) and a regular coffee morning (School C, 6 parents and carers); whilst School B sent out our flier and invited parents to meet with us, no one took up the invitation, and instead we spoke with a mother on the phone. Seventeen parents and carers took part in these discussions in total. With children, given the brief nature of our visits, we relied mainly on observation ( Nind, 2008), supplemented by informal discussions in all three schools and a formal consultation with the School Council in School A (the findings of which are reported under WP1). We had formal discussions with ten pupils in school A and informal discussions with around ten children, alongside our observations of classes and eating occasions, in the other two schools ( Table 5).

**Table 5.** Work Package (WP)4 methods and data collection during the three case-study school visits.

	School A	School B	School C
**Staff**			
*Formal Interviews*	Headteacher Cook and Area Manager	Headteacher	Headteacher Deputy headteacher Chef
*Informal Discussions*	Deputy headteacher Teaching Assistant (TA) TAs × 2 at lunchtime/school council Family Support Worker (FSW)	Teacher Educational Psychologists × 2 TA School Cook	School Nurse
**Parents and Carers**	Yes, group discussion using a flip chart with bespoke coffee morning (n = 10)	No, phone call (n = 1)	Yes, joined existing coffee morning (n = 6)
**Children and young people**			
*Observation & informal discussion Formal Discussions*	Observation ×2 cooking classes (beans/hoops on toast; sausage and mushroom risotto) Observation lunchtimes: two age groups/shifts dining hall older children and young people-college building Observation snack times ×2 age groups Discussion with school council (n = 7)	Participant observation: school bus trip to fast food restaurant (4 children) School bus trip to supermarket (5 children) Observation ×2 cooking classes (pizza bagels) Observation lunchtime: Classroom younger children Classroom tube feeding Observation snack times x2 age groups	Tour of school (incl. Dining hall) and informal discussion (2 children and young people)
**Documents**	Menu School meal numbers Email from Local Authority Trading Company (LATCo) recommendations for special schools	Menu School meal numbers	Menu School meal numbers

Formal (semi-structured) interviews were recorded (with consent) and transcribed in full. Fieldnotes and flip-chart discussions were written up as soon as practically possible after each visit, and each fieldworker’s notes were combined into one account. In terms of documentary evidence, we gathered or were subsequently sent lunch menus from each school. The external catering provider for School A also emailed their team’s suggestions for ‘growing’ Healthy Zones in special schools (the title of the co-produced logic model). None of the three schools we visited had a formal food policy, so we were unable to collect these. Analysis of the school-level data (fieldnotes, notes from meetings, and transcripts) primarily took a comparative case approach ( Yin, 2003) in which schools were compared across relevant dimensions of the whole school food environment (WSFE), combined with thematic and Framework ( Gale *et al*., 2013; Ritchie & Lewis, 2003) approaches (Transcripts of formal semi-structured qualitative interviews have been anonymised and archived).

## Results

Key findings for each WP are described separately below, then summarised in relation to the study’s wider aims.

### Work Package 1

It has been suggested that collaborative logic modelling with stakeholders can be ‘a type of creative practice whose value lies not primarily in the product (model) that is produced, but in the process of iterative reflection that developing a model promotes’ ( Harris *et al*., 2018, p. 1).

**Figure 2.**
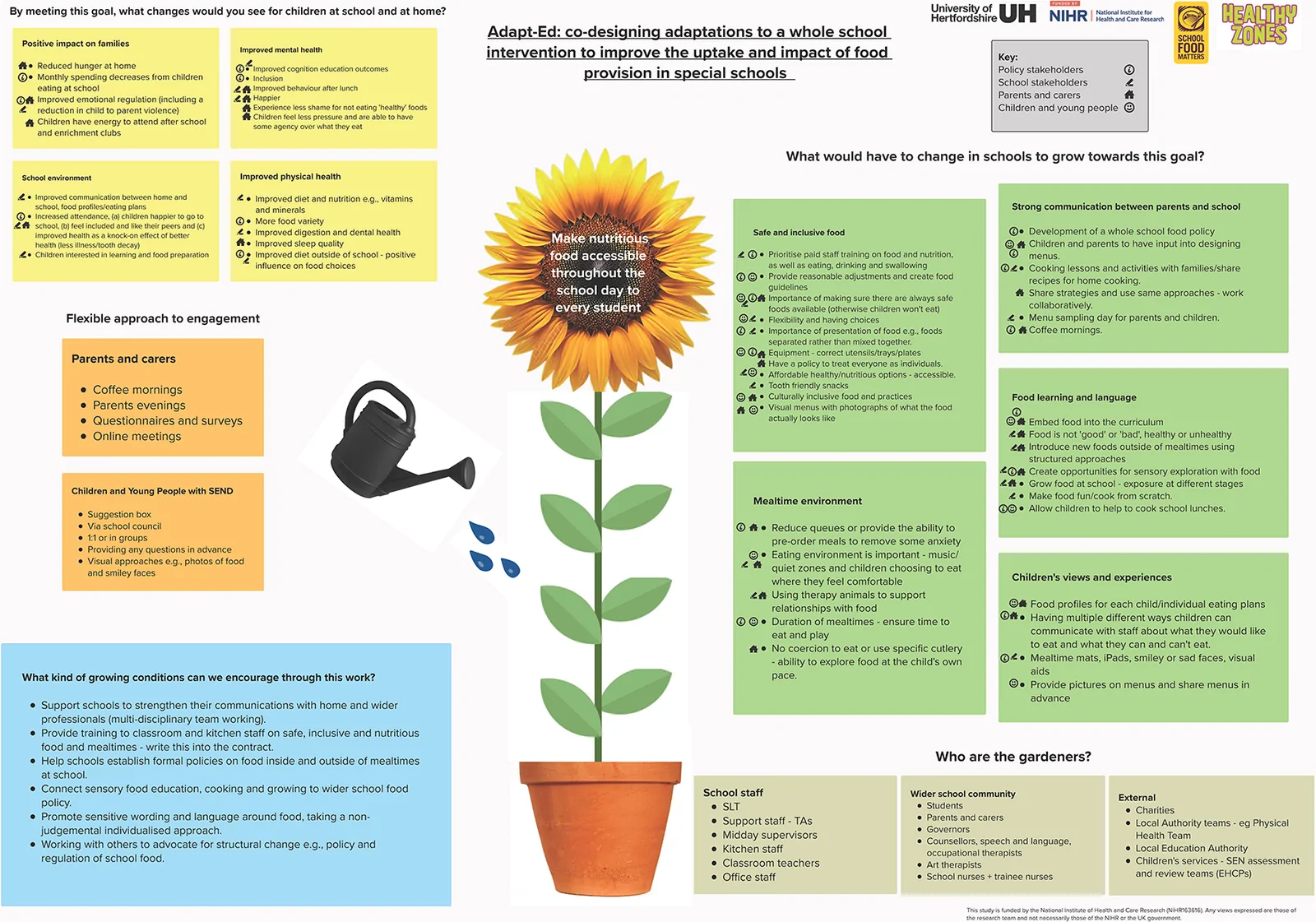
Final collaborative logic model for ‘Adapt-Ed Healthy Zones’. The process of co-producing the final collaborative logic model involved four stages, as follows:
•Stage One: The collaborative process initially took place during the second (of three) meetings with each (adult) stakeholder group separately (1) parents/carers; (2) people working in schools; and (3) people working with schools). Prior to this meeting (stage two), each stakeholder group member was sent a copy of the sunflower model ( Figure 1) to familiarise themselves and consult with colleagues or peers, as appropriate.•Stage Two: During the second meetings, the model shared with each stakeholder group was pre-populated by the research team, drawing on discussions during the previous introductory stakeholder workshops to avoid repetition. The online programme, Mural, was then used to create a more detailed version of the sunflower model. At this stage, three distinct models were generated for each of the three adult stakeholder groups to preserve the specific contributions made by each of the groups. The exception to this was for the ‘who are the gardeners?’ section, that we consulted on and added to during each meeting. By the end of the second group meetings, each group had its own ‘bespoke’ sunflower.•Stage Three: The research team combined these three individual ‘bespoke’ models into one model, using a ‘key’ to indicate the source of the input. As expected, there was some overlap, as well as unique contributions made by each individual group. Relevant contributions from discussions with the children and young people stakeholder groups were recorded in a simplified Framework analysis table ( Gale et al., 2013 ^11^). These were then added to the model during stage two by the research team.•Stage Four: For the third and final online stakeholder workshop, each of the three (adult) stakeholder groups were brought together into one group. The combined model was shared with the group using the programme, Mural. Feedback regarding its completeness and accuracy was overwhelmingly positive. Group discussions suggested that we had broadly reflected the contributions from each of the separate stakeholder groups and that members understood the model. A few correction and omissions were raised, which were noted and later added to the model by the research team, alongside a final check against the simple Framework analysis table.

The simple logic model that we co-produced with our stakeholders is included below ( Figure 2). It identifies the growing conditions, activities, outputs, outcomes and impacts identified by groups as needed to achieve the HZ programme goal of making ‘nutritious food accessible throughout the school day to every student’. As shown by the icons indicating the source of the input, there were many areas in which the stakeholders’ contributions complemented and confirmed each other, as well as areas and concerns that were unique to each group. The model also includes information about how stakeholders want to engage with schools and researchers.

Below, we summarise discussions that iteratively informed and were informed by the co-production processes: (a) views of each group about school food for children with SEND and what improvement would look like and (b) how children and young people, parents and carers and professional stakeholders want to be included in decisions about school meals and in the research. In line with a Framework Approach ( Gale *et al*., 2013) themes include those identified both deductively and inductively – i.e. related to workshop topics whilst leaving room for unanticipated findings.

a) Views about school food and what improvement looks like.

*Children and young people*

**Listen to child voice**: “ *Having choices is important.*” (Young person, face-to-face workshop with SEND youth group, Hertfordshire).

Children and young people with SEND want to be involved in making decisions about school food, including flexibility or options for customising their food. The children and young people we spoke to expressed that they like having choices, and they would like to help to design the menu and give feedback to make ongoing improvements, such as via anonymous suggestion boxes or feedback sheets.

**No surprises**: “ *One bad experience with school food has a lasting impact on diet at home as well as at school*” (Young person, online workshop with SEND youth group, Norfolk).

The children and young people told us that there are high stakes when it comes to food, with bad experiences leaving a lasting impact on the foods they eat and subsequently their overall diet. Some children and young people talked about liking variety, but many told us that they also like to know exactly what to expect.

**Reduce anxiety**: “ *Ordering in advance would be easier than queuing due to being nervous in the line.*” (Young person, face-to-face workshop with SEND youth group, Hertfordshire).

There was an underlying theme of anxiety around food, including queuing up for food such as not knowing if preferred options would be available. They suggested that pre-ordering their meals in advance or the ability to see what is left on the menu on a live board whilst you’re in the lunch queue would reduce this anxiety at mealtimes.

**Be sensitive to sensory needs**: “ *Making sure there is always a quiet place to eat when it becomes too much for me”* (Young person, face-to-face session with School Council in School A). This highlights the importance of the physical environment e.g., somewhere quiet, in the lunch hall with friends or eating in the classroom) and the ability to choose where they would like to eat each day, highlighting a key theme of flexibility and choice. The CYP made it clear that small differences to their lunchtime environment could change their entire experience and ability to eat and enjoy school food.

**Use visual aids**: “ *Have pictures on the menu of what the food actually looks like, so we know what to expect.*” (Young person, face-to-face session with SEND Youth Group, Hertfordshire).

Visual aids were important to all the young people we spoke to. Suggestions included providing photos of the actual food and drink that will be available in their specific school and presenting food attractively. Another suggestion was to serve foods separately for self-assembly if possible (e.g., put sliced fruit on separate plates).

*Parents and carers*

**Take care with language**: *“Living with [a child with] ARFID… I would cut my leg off for my son to try something new. And I think as well, even like the wording ‘healthy’ ‘healthy food’… you all food is healthy…actually all food is good food. And I think actually to a child with ARFID to be told ‘this food’s healthy and this food’s not’… I think it’s the language around food that needs to change. You know it’s not ‘this is healthy’ and ‘this is bad for you’ - it’s food.*” (Parent-carer, online stakeholder workshop).

The language around food and eating was particularly important to this group. They recommended avoiding binary terms such as ‘healthy’ and ‘unhealthy’ that can be a sensitive topic for children with eating and drinking difficulties, including Avoidant Restrictive Food Intake Disorder (ARFID), and taken literally by some children with SEND who may feel foods labelled ‘unhealthy’ shouldn’t be eaten at all. Parents preferred the term ‘nutritious food’ and felt that the availability of ‘safe’ foods (that do not trigger sensory sensitivities) is also important to CYP with SEND as it is fundamental to their mental health and emotional regulation.

**Food is about more than nutrition:** *“If they can’t even engage because they’re hungry, or the food is making them feel upset or worried, then you know – what’s the point?”* (Parent-carer, online stakeholder workshop).

This quote illustrates the anxiety that can be caused by mealtimes, as well as the overlap between hunger and fullness and the ability to concentrate and impact on overall mental health.

**Sensory play and exploration for positive relationships with food**: “ *It should be based on more fun things. Kids like the colour, or the texture, the look – what does it feel like, is it crunchy? It could do something good to your tummy.”* (Parent-carer, online stakeholder workshop).

Parents and carers in this group highlighted the importance of sensory play and removing the pressure around food. Sensory play such as blending Cheerios or counting carrot sticks was suggested to encourage a positive relationship with food.

**The importance of communication between home and school**: *“If there was a way that they could communicate back and be like, you know ‘he was happy in the lunch hall today’ ‘he found this stressful’… that communication pathway without having to contact the school office or having to contact the teacher when you pick him up at the end of the day … just a port of contact would make a huge difference.”* (Parent-carer, online stakeholder workshop).

The group also suggested it was important to strengthen communication between home and school and liked the suggestion of using profiles for each child that could be updated from home with a child’s food preferences/dietary habits.

**Embedding food into the curriculum**: *“Food needs to be seen as a vital part of the curriculum not an isolated part of the day.”* (Parent-carer, feedback after online stakeholder workshop).

Parents and carers felt it was important to include food in the curriculum and that mealtimes should be included within this, as food is central to school life and the ability to learn and concentrate.

*People working in schools*

**Filling bellies vs nourishing bodies**: *“A lot of people don’t really understand what nutrition really means, and they think that filling a belly is more important. That’s their priority is to fill the belly rather than the quality of the food that’s going in.”* (Food Technology teacher, online school stakeholder workshop).

There was an awareness among this group that some children may not have access to a hot meal at home, so it is important that there is a priority for schools to give them this at lunchtime. However within the group there were different views and competing priorities with respect to ensuring a child is fed (so it doesn’t matter what they eat, as long as they’re not hungry) versus providing food that is nutritious.

**Sensory methods**: “ *As simple as you know raw vegetables in, you know like a tomato, a cucumber. Let them touch it, let them feel it, and then explain what’s going to be done with that later on. So later on, that’s going to be made into a salad or something like that. I think if we try and introduce it and make it seem fun, you know that’s a way to get the children involved.”* (School cook, online school stakeholder workshop).

This group emphasised the importance of using sensory methods to introduce new foods and doing this gradually, outside of mealtimes, to remove anxiety and to prevent children refusing to eat altogether.

**Rewards**: *“Rewards don’t have to be food-based, you know, some time playing a board game with your friends. It doesn’t have to be food to be a treat.”* (Teaching Assistant, online school stakeholder workshop).

The group discussed the use of sweets or chocolates as a reward in school but that it’s important that this is avoided for tooth health as children with SEND may struggle to go to the dentist, but also for overall health.

**Improving communication between home and school**: *“I think if we can try and encourage the parents to try and do things at home, and then we follow through with it at school… because I think that is confusing for children as well.”* (School Cook, online school stakeholder workshop).

The group felt there could be improvements in communication between home and school so that the same methods are used across both settings for consistency, and that this would be better for the child.

**Links between lessons and mealtimes**: “ *It’s not particularly a holistic approach across schools. So one department might be doing this, another department will be doing something else… some sort of support or encouragement for everybody to sort of knit together.”* (Food Technology teacher, online school stakeholder workshop).

Additionally, the group felt creating stronger links between lessons and eating occasions e.g., break and lunchtime, would be helpful, alongside increasing mealtime length.

*People working in policy and with schools*

**Resources**: *“I think a key issue is resourcing, making sure that there are the resources in the school, particularly staff resources, to implement this properly. I mean so many of the school meals workers we talk to, they’re really passionate about their job but they say they don’t have enough time, there aren’t enough staff in the kitchen.”* (Union representative, online policy stakeholder group).

This group emphasised the broader context of the education and catering landscape. It highlighted the inadequacy of funding for school meals, with schools operating within tight budgets, schools having to use other funds to supplement school meals, and the importance of campaigning for more school meals funding.

**Visual aids**: “ *Visual resources are really helpful. So they have pictures kind of outlining the different ingredients in the things that are on the menu. So, I think personalised support to kind of develop you know visual resources like that… so not just the menu.” (Public Health Officer, online policy stakeholder group).*

The group also suggested methods for improving children’s access to nutritious food at school, including engaging CYP with SEND in school food using visual resources and interactive methods, as well as co-designing menus with children and their families/carers. This, combined with treating all children as individuals, providing menu sampling days and the option to pre-order meals to reduce anxiety about queuing were suggested.

**Involving the whole school community**: *“Support around developing the best feedback mechanism for pupils. So how do pupils relate to the school or the caterer their opinion on the changes, and their experience of the changes. So whether that’s using you know like interactive… I’ve seen things you know like tablets with interactive smiley face symbols on and things for different foods and whatnot. But that would likely take more time and thought in a special school than mainstream.*” (Policy Officer, online policy stakeholder group).

This group also highlighted the importance of everyone working together for cohesion, including catering companies and their staff, who are often overlooked and not included in the wider school community. They also felt that pupils feedback was important to ensure that any mealtime changes were right for them.

**Communication between school and home**: *“Having the individual conversations with the parents to find out what they already do at home… and I think that’s really important… So if a child is already getting a hot meal at home at dinner time, then their focus is not on getting them a big lunch. It’s about getting enough to sustain them through the day in order to get home to have the dinner.”* (Parent-carer representative, online policy stakeholder group).

Similar to our other stakeholder groups, the group felt that it was important to strengthen the communication with home and school so that approaches are consistent and the priorities are the same, to improve the child’s eating and overall health.

b) How children and young people, parents/carers and professional stakeholders want to be involved in decisions about school meals and in the research.

*Children and young people* said they like to provide input in a variety of ways e.g., feedback sheets with ‘emojis’ (smiley or sad faces), using a menu core board or communication book, suggestion boxes, typing answers on a computer, speaking out loud, handwriting or using picture cards. The groups also felt having questions in advance and time to prepare answers can be important.

*Parents and carers* told us that using a range of methods to engage parents is important and to be creative in order to reach parents and carers who do not do the school drop off if their child gets transport to and from school (may also live far away).

*People working in schools* suggested engaging wider school staff (e.g., teaching assistants) in data collection because they work 1:1 or in small groups with the same children throughout the year.

*People working in policy/with schools* suggested that schools maintaining good relationships with parents is very important, finding out what parents do at home and the priorities for the children’s eating, e.g., if they need a hot meal at school because they won’t get one at home. In addition, the group indicated for us to be careful with the wording of the research and not to single out a particular group, to be inclusive of all children with SEND.

### Work Package 2

Scoping review 1 - Healthy eating interventions for children and young people with Special Educational Needs and Disabilities: Initial searching of electronic databases yielded 1900 reports, of which 59 were retrieved for full text review. A total of 19 studies satisfied the inclusion criteria ( Table 2) and underwent data extraction. Interventions were predominantly targeted at CYP with autism but also included those with Attention Deficit Hyperactivity Disorder, Down syndrome, Spina Bifida, Cerebral Palsy and Intellectual Disability. Healthy eating interventions were classified according to their primary focus and sub-type.

Of the nineteen included studies, ten evaluated education-based interventions ( Nury et al., 2022), with six of these focusing on general health promotion (e.g., emphasising the benefits of both healthy eating and physical activity) and four targeting food and nutrition education to improve nutrition knowledge. The remaining nine studies assessed behaviour-based interventions ( Al-Beltagi, 2024), including four that focused on the sensory characteristics of food (e.g., texture, shape, and appearance) and five that targeted the food or home environment (e.g., the use of rewards, modifications to meal options, and caregiver encouragement).

Education-based interventions primarily aimed to improve dietary intake, nutrition knowledge, food literacy, cooking skills, and healthy lifestyle behaviours through nutrition education, counselling, mentoring, and health promotion activities. Behaviour-based interventions focused on increasing food acceptance and variety, reducing food selectivity and eating aversions, improving food choices, and enhancing dietary intake through sensory exposure, environmental modifications, rewards, encouragement, and behavioural shaping techniques. Outcome measures included dietary intake, food acceptance, variety, or preferences, food selection, eating behaviours, and nutrition knowledge or practice. Overall, the interventions targeted both knowledge-related determinants of healthy eating and behavioural factors influencing food acceptance and consumption.

Outcome measurement varied substantially across the included studies. A notable limitation was the lack of validated assessment tools, with many studies relying on self-report measures and open- or closed-ended questionnaires. Consequently, the validity of the findings and the ability to compare intervention effectiveness across studies may be limited. The full results of the scoping review have been written for publication (Denyer et al., forthcoming).

Scoping review 2 - Measuring diet and eating behaviours in children and young people with special educational needs and disabilities: Initial searching of electronic databases yielded 1501 articles, with 101 articles taken forward for full text review. A total of 72 studies satisfied the inclusion criteria and examined children with a range of special educational needs and disabilities, including autism, Attention Deficit Hyperactivity Disorder, Cerebral Palsy, Intellectual Disability, and Down syndrome (Inclusion exclusion criteria are shown in Table 3).

Among the 72 included studies, over 25 different assessment tools were identified. Dietary intake was evaluated using a variety of approaches. Self-reported methods included Food Frequency Questionnaires, 24-hour recalls, and weighed food records, while objective techniques comprised direct mealtime observation, biological markers of nutritional intake, and digital or mechanical tools for quantifying food consumption. For measuring eating behaviours, parental reports, standardised eating behaviour questionnaires, and food frequency questionnaires to examine associations between diet, behaviour, and developmental conditions at a single time point.

Existing tools were insufficient to capture the full complexity of dietary intake and eating behaviours among children with SEND. Research in this field has largely concentrated on food selectivity, with assessments predominantly relying on parent-reported measures such as questionnaires and food diaries. Furthermore, there was minimal attention given to the contextual and environmental factors in which eating behaviours and challenges occurred.

Taken together, the complex and diverse nature of feeding difficulties in SEND, coupled with limited suitable assessment instruments—particularly for children who are non-verbal or have intellectual disabilities—makes meaningful comparison across populations difficult. The full results of the scoping review have been written for publication (Ludlow et al., forthcoming).

Overall, our scoping studies found that there has been a focus on addressing and measuring autistic children feeding difficulties and diet, and comparatively less on children with motor difficulties and/or intellectual impairment. Furthermore, as a recent study has confirmed, there is a scarcity of research assessing children’s eating behaviours and dietary intake specifically within a school environment ( Spence et al., 2026), however, this is vital to understand as children typically consume around half their total daily energy intake at school.

### Work Package 3

To explore the relationship between school characteristics and the take up of FSM, we conducted (a) analysis of routine publicly available administrative data; (b) an online school survey; and (c) an ‘audit’ of special schools’ websites.

a) *Secondary analysis of routine publicly available data:*

*i) Descriptive analysis of free school meal eligibility and take up in the East of England:* Our analysis of EoE schools found that special schools have around twice the proportion of children *eligible* for FSM as other types of school ( Table 6). However, we found that *take up* of FSM in special schools lags behind other school types in EoE, with over one-in-four FSM-eligible pupils (27.4%) and nearly one-in-four infant pupils (24.5%) not taking up their entitlement ( Table 7). The gap for infant FSM is especially stark when comparing special schools (75.5% take up), primary with SEN provision (84.7%) and mainstream primary (86.9%). Take up in Special and Primary schools tends to be slightly lower in EoE compared to the rest of England, and slightly higher for Secondary schools.

**Table 6.** Descriptive statistics of pupil and school characteristics in the East of England. Key point: Special schools in the East of England have around twice the proportion of children eligible for Free School Meals as other types of school.

%, unless stated	Special schools	Primary with SEN Unit or Resourced Provision	Secondary with SEN Unit or Resourced Provision	Pupil Referral Unit (PRU) with SEN provision	Primary mainstream only	Secondary mainstream only
*Pupil characteristics, %*						
Means-tested Free School Meal (FSM) eligibility	44.5	24.5	22.1	34.1	19.4	20.8
Male	73.2	51.4	51.5	57.6	51.0	50.6
White British	73.4	66.0	68.1	79.3	71.6	67.3
Black	7.6	6.3	6.1	5.6	5.8	6.4
South Asian	7.0	6.5	6.2	2.2	5.8	6.0
Other ethnicity	15.6	24.1	18.7	25.1	19.1	19.7
English as alt’ language	8.1	5.9	1.3	*	6.1	3.0
*School characteristics, %*						
Conurbation	14.0	10.2	8.3	9.5	8.4	12.3
City or Town	71.1	72.9	65.0	85.7	49.0	67.9
Rural	14.9	16.9	26.7	4.8	42.6	19.8
Academy Converter	30.7	32.2	63.3	57.1	30.2	58.4
Academy Sponsor-led	11.4	25.4	26.7	0.0	15.9	23.1
Community	30.7	35.6	0.0	0.0	30.3	1.3
Foundation	9.6	0.0	0.0	0.0	0.0	0.0
Free School	17.5	0.8	5.0	0.0	1.8	10.1
Mean number of pupils per school	142.3	376.0	1205.9	37.7	155.5	1150.1
**N Schools**	114	118	60	21	1843	308

**Table 7.** Take up of Free School Meals (FSM) by school type, % of eligible pupils. Key points: Take up of Free School Meals in the East of England is lower in special schools than other school types, The gap is especially large when comparing Universal Infant FSM take up in special schools with primary schools with SEN provision or mainstream primary schools.

	Means-tested FSM (all ages)		Universal Infant FSM	
School Type	East of England	Rest of England	East of England	Rest of England
Special Schools (All)	72.60	75.96	75.50	78.32
Primary Resourced or SEN Unit	77.18	79.92	84.66	86.55
Secondary Resourced or SEN Unit	78.63	74.38	NA	NA
Primary Mainstream only	79.62	81.00	86.89	87.85
Secondary Mainstream only	76.90	75.71	NA	NA

Note: Sample Ns as per Table 6.

*ii) Multi-variate analysis of FSM take up (England* ^12^ *):* Special schools have similar take up of means-tested FSM compared to other primary schools with the same pupil demographic characteristics, location, and governance type ( Table 8). Within non-special schools serving the same age groups, there is no significant difference in take up between those with a SEN Unit or Resourced Provision and mainstream-only schools. However, special schools have considerably lower Infant FSM take up than other schools (by 6–7 ppt). Aside from this, very little variation in FSM take up is explained by demographics, school type or location. To the extent that take up of FSM can proxy for a successful school food environment, this suggests that stratifying a future randomised trial only based on routinely collected would not improve precision, and that information about policy and practice within schools will be essential for this purpose.

**Table 8.** School-level predictors of Free School Meal (FSM) take up. Key points: Other things equal, special schools have similar take up of means-tested Free School Meals (FSM), but considerably lower Universal Infant FSM take up than non-special schools. However, very little variation in FSM take up is explained by demographics, school type or location.

	(1)	(2)	(3)	(4)	(5)	(6)
	All Schools		Special Schools		Schools with SEN Unit or Resourced Provision	
	Means- tested FSM	Universal Infant FSM	Means- tested FSM	Universal Infant FSM	Means- tested FSM	Universal Infant FSM
***Special Educational Needs (SEN) Provision: Relative to Special Schools***						
Primary SEN or resourced	−0.441	6.354***				
	(1.145)	(0.721)				
Secondary SEN or resourced	−9.903***					
	(1.301)					
Primary Mainstream	0.767	7.283***				
	(1.053)	(0.675)				
Secondary Mainstream	−10.249***					
	(1.093)					
***Relative to Rest of England***						
East of England	−0.866**	−0.621**	−2.848	0.337	−0.479	−0.796
	(0.413)	(0.260)	(2.064)	(2.603)	(1.476)	(0.999)
***Pupil Demographics***						
% FSM Eligible	−0.076***	−0.054***	−0.052	0.006	−0.030	−0.007
	(0.010)	(0.006)	(0.057)	(0.074)	(0.033)	(0.021)
% Male	−0.073***	−0.020	−0.080	0.106	0.016	−0.055
	(0.016)	(0.017)	(0.066)	(0.100)	(0.067)	(0.084)
% Black	−0.007***	0.001	0.065	0.145*	0.005	0.033
	(0.002)	(0.001)	(0.065)	(0.085)	(0.050)	(0.032)
% South Asian	−0.004**	0.022***	0.072	−0.051	0.061**	0.033
	(0.002)	(0.006)	(0.065)	(0.078)	(0.029)	(0.021)
% Other minority ethn’	−0.005***	0.005	−0.007	−0.087	0.057*	0.016
	(0.002)	(0.005)	(0.072)	(0.096)	(0.033)	(0.022)
% English Alt’ Language	−0.017***	−0.021***	−0.020	0.067	−0.151**	−0.037
	(0.006)	(0.004)	(0.042)	(0.048)	(0.061)	(0.044)
Share Primary Age (0–1 scale)	0.774	2.423**	5.632***	9.703***	−12.718	0.057
	(1.362)	(0.954)	(1.933)	(3.088)	(7.944)	(4.562)
***Location: Relative to Conurbation***						
Town or City	−2.589***	−2.598***	−2.689*	−4.231**	−0.256	−2.186***
	(0.316)	(0.206)	(1.569)	(1.953)	(0.994)	(0.674)
Rural	−1.385***	−2.020***	−4.432*	−3.308	0.437	−0.861
	(0.399)	(0.269)	(2.633)	(3.320)	(1.649)	(1.187)
***School Type: Relative to Community School***						
Academy Converter	1.597***	−0.564***	1.329	−2.888	0.635	−1.069*
	(0.299)	(0.183)	(1.559)	(1.917)	(0.956)	(0.624)
Academy Sponsor-Led	2.605***	−1.276***	2.387	−2.133	0.656	−1.622**
	(0.438)	(0.287)	(2.610)	(3.453)	(1.236)	(0.824)
Foundation School	−4.083*	−1.370	−3.805	−2.588		
	(2.188)	(1.429)	(2.478)	(2.867)		
Free School	5.899***	1.449**	5.397**	−4.016	1.918	5.704*
	(0.805)	(0.603)	(2.451)	(3.482)	(3.496)	(3.210)
Number of Pupils	0.005***	−0.003***	−0.004	0.009	0.003*	−0.002
	(0.001)	(0.001)	(0.009)	(0.010)	(0.002)	(0.002)
Any Boarding Pupils	5.576**	−5.255**	5.900**	−4.404	5.996	
	(2.211)	(2.148)	(2.945)	(4.233)	(12.736)	
N schools	20753	16158	1028	653	1920	1211
R^2	0.037	0.055	0.045	0.066	0.033	0.033

Note: Standard errors in parentheses; ***,**,* indicate statistical significance at 1%, 5% and 10% levels respectively. Columns 3–6 additionally control for the presence of Resourced Provision, a SEN Unit or both.

*b) Online school survey (East of England)*: 33 schools responded, including 16 special schools and 17 with a SEN Unit, Resourced Provision or Special Classes. Compared to non-responding schools, responding schools are somewhat positively self-selecting, with higher take up of means-tested FSM and UIFSM recorded in routine data ( Table 9). Despite their apparent positive self-selection, only four responding special schools (25%) and one other school (6%) have a written school food policy; and 7 and 6 (44% and 35%) respectively have a named member of staff or governor responsible for school food ( Table 10). Both school types had equal numbers with an external catering provider (i.e. local authority or private company) and in-house provision. With respect to other aspects of the whole school food environment (WSFE), all responding schools (n = 33) include cooking, and most (n = 24) include gardening, in food education activities. Other food education activities include community outings and using sensory approaches to encourage tasting or exploring of new foods. Schools also reported which aspects of the school dining environment may be important for school meal take up. Notably, pupils’ sensory issues were mentioned as a key factor, including: difficulties with noise and large groups of people; limited or specific food preferences (e.g. due to ARFID); how food is plated; and/or aversions to certain tastes, smells and textures. Other factors include portion size, cost and variety of meals; dining space/capacity; and the number of staff to support pupils with complex needs (e.g. gastrostomies).

**Table 9.** Characteristics of responding and non-responding schools in survey sample frame. Key point: Compared to non-responding schools, responding schools have higher take up of means-tested Free School Meals (FSM) and Universal Infant FSM recorded in routine data.

	Special Schools		Schools with SEN Unit, Resourced Provision or Special Classes	
	Responding	Non- Responding	Responding	Non-Responding
Number of Pupils	148	140	461	703
Town or City (%)	68.8	70.0	30.8	71.2
Rural (%)	25.0	14.0	46.0	18.4
FSM Eligibility (%)	48.0	44.2	17.4	23.2
Means-tested FSM take up (%)	73.3	73.0	82.2	73.9
Universal Infant FSM take up (%)	93.6	74.0	91.3	77.9
Paid take up (%)	58.7	*Not available*	58.2	*Not available*
N schools	16	98	17	210

**Table 10.** Online school survey self-reported food-related policy and practice (%) in responding special schools (N = 16) and mainstream schools* (N = 17), by school type. Key points: Responding schools are equally likely to have in-house catering as an external provider. Few responding schools have a written school food policy, and a minority have a named member of staff or governor responsible for school food. Few responding schools are currently receiving external support or are involved in a health-related scheme or accreditation programme, but a majority of responding schools are interested in receiving additional support to improve school food or in taking part in a future study trialling the adapted Healthy Zones programme.

	Special	Other		Special	Other
***Type of school catering provision***			***School provides food education activities***		
In-house catering	50	47	Cooking	100	100
External catering	50	47	Gardening	82	82
***Rating quality of food and service***			***Time available for lunch and play***		
Excellent	31	41	15–29 mins	13	6
Good	25	18	30–44 mins	13	18
Fair	44	35	45–59 mins	31	59
Poor	0	6	60–89 mins	44	18
Very Poor	0	0			
***Have parents spoken about getting vouchers to buy a packed lunch instead of Free School Meal FSM?***			School meals and packed lunches generally eat together	94	94
Yes	13	0	No staggering of meals at all	25	6
Don’t Know	81	100	Staggering by need/discretion	19	12
No	6	0	Staggering by year-group/class	56	82
Parents choose children’s meals	38	23	***SEND support for pupils to eat at lunchtime***		
Children choose their meals	38	0	Type of food (e.g. dietary restrictions)	100	88
It depends on individual student needs	25	76	Eating in particular environments	100	88
School has written School Food Policy	25	6	One-to-one physical support	75	52
School has named staff member responsible for school food	44	35	Specific SEN difficulties or discomfort (e.g. sensory or food phobias)	69	59
School has named governor responsible for school food	6	0	Currently receiving external support to improve school food	25	6
			Currently part of school food and/or health related scheme or accreditation programme	19	12
**N responding schools**	16	17			

^*^ Mainstream schools with Special Educational Needs (SEN) Unit, Resourced Provision or Special Classes. Categories may not add up to 100% if schools did not answer, did not know, or provided an answer not shown here.

The main challenges, as reported by schools, when providing food for or feeding children with SEND are not having enough staff, a lack of appropriate space or limited facilities, and limited funding. Other reported challenges included the type and extent of support that some children require, such as medical or physical mobility needs, swallowing, choking (dysphagia), allergens and providing specialist medical diets (e.g. via feeding tube or blended food). Some schools also reported that behavioural issues (e.g. oppositional behaviours), related to specific types of SEN, are more likely to occur due to sensory overwhelm, frustration from queuing, a lack of control and/or dislike of the food available, which can also affect the other pupils.

Whilst numbers are too small to draw firm conclusions, exploratory descriptive analysis suggests that the following factors (unobserved in admin data) *may* be associated with school meal take up: meal cost, time available for lunch and whether the school is part of an external food-related scheme or accreditation programme. There is no relationship between take up and catering provision (external vs. in-house) or whether schools have a food policy.

A majority of schools (n = 27, 82%) said they were somewhat or very interested in receiving additional support to improve their school food. Thirty schools (91%) stated that they would or might be interested in taking part in a future study trialling an adapted Healthy Zones programme. Schools responding ‘it depends’ (n = 2) expressed concerns about funding during the intervention and beyond the first year, staff time necessary to implement the intervention, and what the impact would be on pupils’ eating habits.

*c) School website audit (East of England):* An ‘audit’ of EoE special schools’ websites (N = 114) explored what other relevant information could be gleaned from these. Only 11 schools (10%) had a school food policy available online. Seventy-nine schools (69%) had identifiable catering providers, of which 50 (63%) were ‘external’ and 29 (37%) were ‘in-house’. Lunch menus were available online for three-quarters of schools (n = 87, 76%). Given the availability of menus online, they are a potential resource for a future study (e.g. analysis of nutrition and adherence to school food standards). However, as noted below (WP4: Table 11), menus may not accurately represent foods available.

**Table 11.** Comparing special school contexts (N = 3) using a Whole School Food Environments framework ( Moore *et al.*, 2023).

WFSE dimension	Similarities between schools			Differences between schools
	What	Detail	Illustrative quotes	
**Educational philosophy and approach**	‘Equity’ and care prioritised	- Children who are not hungry better able to regulate behaviour and learn - Children need lunch as may not eat at home due to poverty	‘[m]ental health goes along with having a full tummy doesn’t it.’ (Head, School B) ‘if you look at the catchment area we serve […] iit’s not just about the entitlement to a free school meal … but it is the only meal they bloody well get’ (Exec Head School C)	**N/A**
**Policies & leadership**	Need for flexibility, to meet individual needs	- None has food policy – resistant - Food and eating needs as per EHCPs	‘We are very much … it’s about the individual youngsters, so often with policies it’s about that generic kind of blanket approach isn’t it? (Head, School A)	**N/A**
**Food provision**	Food recognised as about more than nutrition - sensory, control and wellbeing	- All cooks present/ serve foods in ‘appealing’ ways (e.g. raw/separated) - Packed lunches & snacks high levels of pre-packaged processed foods	‘it’s that real core understanding that for some young people […] eating their chicken dippers – it’s soothing, it’s a self-regulation strategy, and it’s incredibly important to their mental health and wellbeing’ (Head, School A) ‘Children eat with their eyes’ (Chef, School C)	**Catering arrangements:** Two schools (B and C) with internal caterer *(but not LatCo, School A)*: -provide ‘bespoke’ lunches (these are “off menu”) -children choose own meal at morning registration ‘People need to ask “what children *like* to eat, not just what they *can’t* eat” [FN Teacher, School B]
**Physical setting**	Sensory environment acknowledged to shape eating	- Some or all children eat in classrooms	‘the main dinner [hall] is a lot noisier [than the packed lunch area], so he will … it’s just not the food is it, there’s all … so many other issues of why these children [do or do not eat school meals]’ (Parent of an autistic child in mainstream school, School A)	**School size/layout** – rural School A with large footprint – food travelling distances to classrooms in ‘hot boxes’ can spoil: ‘I think for children that have special needs and are visual and stuff, my personal opinion on that is the way the dinner is served here isn’t as good quality by the time the children get it’ (Cook, School A)
**Food education/ activities**	‘Food ‘skills’ recognised as fundamental to independence and wellbeing (important aspects of special education)	- Regular cooking in ‘enabling spaces’ to explore foods and eat (or not) - Food shopping and other community activities (e.g. restaurants) - Gardening and growing food more irregular (staffing)	‘[The Ed Psych said] during Covid many children regressed. Parents struggled. G’s mother [a lone parent] was at her wit’s end. He refused to wear clothes. He took over the house, took control of the remote control and the phones, ate all the food in the cupboards. They did a home visit. He could not be in school but they took him out on the bus [to MacDonalds] – they masked up, he sat at the back. He had to get dressed for that’. [FN Drive Thru, trip, School B]	**N/A**
	Food used as a medium for learning	- Sensory activities/approaches throughout - Mealtimes as pedagogical - children (supported by TAs and MSAs) encouraged to use ways of communicating - Food ‘very motivating’ for some - generally less-healthy foods used as incentive/ reward	‘there are some of the base classes that do work that way [using food as a reward] – that’s what motivates the children’ (assistant Head, School C).	
**Parental/child engagement**	Children’s eating recognised as embedded in family routines & relations	- Parental engagement vital but difficult; generally 1:1 on phone or note in ‘home-school’ book/bag - Children’s ‘say’ mostly limited to choice of foods	‘[M] ost parents want us to change things but find it very difficult to put those rules, boundaries and limitations in themselves - so that is a major hurdle.’ (Head, School B) “we could support [children] a lot more with better diet. But it is starting with parents, isn’t it? (Exec Head, School C) ‘because of our big catchment obviously most of our children are being bussed in, we’re not seeing parents on a regular basis (Assistant Head, School C)	**Participation in an accreditation scheme:** - Only School C formally consulted children (school council) - prompted by ‘Healthy Schools’ - Only School C had workshops for parents on healthy eating and lunchboxes - prompted by ‘Healthy Schools’ **Area affluence:** - Schools A and C had regular coffee mornings but School B most parents in paid employment - relatively affluent area

^1^ What is equal (same or uniform) differs from what is equitable (fair or just).

Of the 114 special schools included in the website audit, 16 also completed the online school survey, which asked about catering provision (internal vs. external) and whether the school had a written school food policy. There was only one discrepancy in the type of catering provision recorded across these two datasets. However, of the four special schools that stated they had a written school food policy via the online school survey, only one of these schools had made the policy publicly available on their website. This suggests that conducting an audit of school websites is not sufficient to ascertain whether ADD 16 and 17 to bottom right two cells have a written school food policy.

### Work Package 4

The ADAPT guidance ( Moore *et al*., 2021: 4; Pfadenhauer *et al*., 2017) suggests that, in making decisions on the nature and extent of adaptation needed to achieve a good fit between an existing intervention and new context, the ‘use of an existing theory or framework (or elements of these) to structure thinking about context can be useful’.

In analysing the ethnographic data, we compared school food contexts across broad dimensions of the *whole school food environment* (WSFE) that, following Moore *et al*.’s (2023) framework, we summarised as: food policies and leadership; food provision; the physical setting; food education/activities: parental and child engagement in school food (see also Bryant *et al*., 2023). To these established WSFE dimensions, we added another aspect of school-level context: ‘ *educational philosophy and approach’*, that emerged during our analysis as fundamental to understanding the provision of food for children in special schools, in that it underpinned schools’ approaches in the other areas. Table 11 summarises the comparison of schools’ approaches according to these dimensions, identifies aspects of context that were similar as well as those that seemed to make a difference to school food provision and practices, and provides brief selected quotes from interviews to illustrate key points. In comparing school contexts across the WFSE dimensions, as Table 11 shows, we found many similarities and some differences:

In terms of the ***educational philosophy and approach***, in all three schools there was an emphasis on schools’ role as providers of ‘care’ as well as ‘education’. Schools’ ‘ethic of care’ included recognition of the needs of individual children and the (often challenging financial) circumstances of many families - caring ‘for’ and caring ‘about’ ( Tronto, 1998). This entailed an attention to ‘equity’ as opposed to ‘equality’, wherein what is fair or just was prioritised over what is the same. In terms of food this meant understanding that ‘one size does not fit all’ and that flexibility is needed in the application of rules. Priority was also placed on children eating and drinking something at school, so that they were able to regulate their behaviour, and because they might not eat at home.

The three schools were also similar with respect to ***policies and leadership***; none had a school food policy and in each there was some resistance to the idea, with emphasised placed on children’s individual needs, as articulated in their EHC Plans, and the inappropriateness of ‘blanket’ rules. An exception to this was food safety: all schools had clear rules around this, for example, the refrigeration of food from home or not reheating previously cooked food. Unsurprisingly, then, none of the staff had food leadership in their roles, however some health was in the remit of some senior leaders.

In terms of ***food provision***, in all three schools there was recognition from teaching, catering, support staff and parents and carers that food is about much more than nutrition, particularly for children with sensory sensitivities, and very important for children’s sense of control and wellbeing. However *catering arrangements* made a difference to how flexible catering staff could be. In all three schools, Cooks were able to and did amend *presentation* of food, serving foods in ways they hoped were more appealing for some children e.g. raw or separated. However, in terms of food *provision,* in the two schools in which the cook was employed by the school (Schools B and C), pupils were able to choose meals, and cooks provided completely or partially bespoke meals, but this did not happen in School A in which the catering was provided by a LATCo. Packed lunches and snacks brought by pupils were in all three schools observed and/or said to be high in pre-packaged and processed foods.

In terms of the ***physical setting***, the importance of the sensory environment was acknowledged to shape children’s eating and in all three schools some or all the children ate lunch in classrooms rather than the dining hall. However, the school size and layout seemed to impact on the quality of the food, because food travelling in hot boxes to classrooms some distance away could spoil (e.g. chips could get cold and soggy), which was particularly challenging for children with sensory sensitivities. Hence, whilst the need for a calmer eating environment was acknowledged by all, there were challenges and trade-offs, particularly for the rural school with its larger footprint.

As ‘outsiders’ or ‘strangers’ ( Schütz, 1964) to special education settings, we were struck by the centrality of ***food and food education/activities*** in the curricula of the special schools we visited. In all three schools, reflective of the purposes of special education, ‘food skills’ were recognised as fundamental to independence and wellbeing. Pupils in all three schools took part in regular cooking activities in ‘enabling spaces’ in which they were invited to explore and/or eat food. In all three schools, children took part in food shopping and other ‘community’ activities (e.g. making shopping lists, doing food shopping and making trips to restaurants). Whilst there was provision for growing food in all three schools this was less well supported, with a neglected balcony in one school and unused polytunnel in another due to staff led turnover. Food was also used as a medium for learning in all the schools, ranging from sensory play to the (more controversial) use of (less healthy) food as a reward, as food was seen as very ‘comforting’ or ‘motivating’ for some children.

Finally, with respect to ***parental/child engagement in school food***, the embeddedness of children’s food and eating within family routines and relations ( Albon, 2007; O’Connell & Brannen, 2016) was widely recognised and emphasised by staff. This meant schools saw the need for and value of involving parents in decisions about children’s eating, with most engagement 1:1 through a phone call or note in the home/school book or children’s bags. Because of wide catchments and use of transport, schools found it difficult to encourage parents to take part in group activities. However, two of them (Schools A and C) held regular coffee mornings whilst the other school (B) in a more affluent area found this did not work, as a large proportion of parents were in paid work. With respect to children and young people’s involvement in school food, it was quite striking, given the emphasis on children’s individual needs, that they (and parents) had only been formally consulted about school food in one of the schools (School C). This was prompted by the school’s participation in the Healthy Schools scheme. When asked about their views of school food as part of this process, the children had shared lots of ideas about what they would like to see on the menu, including fruit smoothies. The parents we talked to in Schools A and C, and the children in the School Council (School A) had lots of ideas about how to improve food at school, and suggestions for how childen might be supported to share their views. These are covered above (WP1) and in the next section on ‘school staff and parents and carers’ views, as well as in the draft sunflower logic model ( Figure 2).

### School staff, parents and carers’ views on acceptability, impact and feasibility

In addition to comparing school contexts in terms of their WSFE, we spoke with parents, carers and staff about their views on opportunities, challenges and acceptability of the intervention; what improvement would look like and the impacts we would see; and the feasibility and practicalities of delivery and collecting data. These are summarised below.

***Opportunities, challenges and acceptability.*** The members of staff we spoke with in all three schools believed food and eating played an important role in children’s health and wellbeing, and Headteachers in all three schools expressed interest in taking part in a future intervention.

Schools recognised they had a responsibility and opportunity to support healthy food and increased dietary diversity. Headteachers also mentioned that eating at home could be difficult and parents’ anxiety that children ate could transfer onto children. However, their experience was that the school environment could relieve some of this stress:

*‘[B] ecause we’re not their mums and dads, and you know we don’t have that same kind of parental worry around ‘Oh my child needs to eat and it’s my fault if they don’t’ – do you know what I mean? […] I think that just takes a little bit of pressure out of the whole situation, and that I think is why sometimes we see young people more open to trying different things in the school environment’* (Headteacher, School A).

However, staff recognised that pressuring children with highly restricted diets to try or eat foods risked them stopping eating altogether. The need to take a case-by-case approach was mentioned, and a diversity of methods for introducing new foods was observed. This included using ‘ *taster plates’* at lunchtime, to allow ‘ *room for growth’*, even where children had packed lunches, or were mostly fed by gastronomy. However, there were different views; some suggested it was better to avoid the introduction of foods at mealtimes at all, and stick to other times, whilst others mentioned being confused about what was best and needing access to specialist advice. The need to consult with and involve parents was mentioned by staff in all three schools, as were the challenges of this, given what one Head called ‘ *fixed mindsets’*, as well as the practical constraints of wide catchment areas and some parents not having cars. Parents highlighted the stress of mealtimes at home that at times triggered violence, and having a lack of time and energy, and other priorities.

***What improvement would look like look like and the impacts we would see.*** Staff in two schools mentioned improvements they would like to see to the WSFE, specifically *a salad bar that children could select food from* (only one school had this). More than one parent mentioned the importance of children *being able to see food*, so they knew what to expect – visiting the kitchen or seeing photographs of actual meals. Some staff seemed to want to see an improvement in terms of the nutritional quality of the *foods children brought to school* to eat, in some cases appearing dismayed at pre-packed highly processed contents of many packed lunches and snacks, but also keen to emphasise they ‘understood’ the challenges and were ‘not judging’.

Parents also said they wanted to see an increase in the variety of foods their children ate, especially nutritious foods like fruit and vegetables, and to have less stress around food and eating at home. However, some were sceptical about children’s capacity for change, whilst staff emphasised the need for parents to be ‘on board’, and both groups talked about the slow nature of progress, ‘small steps’ and not expecting ‘miracles’. The Head in School B said,

*‘We don’t tend to target children – “they will be eating carrots by this time next year” - because we’d really shoot ourselves in the foot if we did that’* (Head, School B).

In terms of where impacts of improved school food could or would be seen, *being able to access a school meal that they wanted to eat* at lunchtime (in two of the schools) was seen to have an ‘immediate’ impact on children’s *happiness.* School B had recently introduced repeated popular meals daily, with one changing option, and children ordering at morning registration. The Head said that as a result there had been *fewer behavioural incidents* at lunchtime and ‘ *it makes playtime happier because you haven’t got children still indoors in crisis because they didn’t get what they wanted for dinner’.* And in School B, that had a ‘smiley’ new Chef who encouraged children to enjoy school meals, the Executive Head said the children *‘have been much happier after their school lunches’.* Staff also noted the impacts of sugar and additives on children’s *behaviour*, ‘especially’ for children with attention hyperactivity disorder (ADHD), implying there could be potential for improved behaviour with a reduction in processed and sweetened foods and drinks.

The Head of School A also said impacts of giving children what they liked to eat for lunch were that it *‘saves money, saves time’,* whilst the Cook said there was much *less waste.* The Chef in School C mentioned the results he would see from an improved WSFE would be an increase in *numbers having school meals* and consequently a reduction in ‘ *cost per meal’.* (It is important to note with respect to this potential indicator that figures collected from the schools on take up of paid and FSM for the weeks we visited diverged from the administrative FSM census day data; this suggests it could be a mistake to rely on administrative data to examine impacts on uptake at individual school-level).

Potential longer-term impacts on the diversity of children’s diets and eating behaviours at school and home were also mentioned. The Head in School A described using ‘tasting plates’ with a boy who’d had a very restricted diet, and as a result of gradually introducing new foods at school, he could eventually eat school meals. This was said to have had impacts on parents too:

*‘it took from the age of 12 through till he was 19, but when he left us he was eating full meals, a variety of meals, most of the meals that was offered on the menu. And you know as a result his health improved … .. once they (the parents) saw some success at school, that gave them some confidence to try different things at home’* (Head, School A).

***Feasibility and practicalities of delivery and collecting data.*** According to a teacher in School B, feasibility depends on the intervention ‘ *slotting in’* to what the school is already doing. The Head of School B suggested using data already collected by teaching staff on children’s ‘engagement’ in learning: *‘that’s a big part of our lives, looking at engagement.*’ She also suggested monitoring changes documented on children’s Education, Health and Care Plans (EHC Plans) as ‘ *if there’s a problem with eating then most of them would have something on their education health and care plan about it’.* The Head in School A also suggested we could draw on data staff already collected on emotional regulation and behaviour, alongside researchers monitoring food through ‘food diaries’. However, she noted the importance of contextualising data about what children eat, as ‘who they sit with’, for example, may influence what they eat. The Executive Head (School C) noted it would be ‘difficult’ to monitor progress as children have very different starting points. One Teaching Assistant (TA) told us TAs would want to support the study by collecting data, since they did the job because they cared about children’s wellbeing. Asked about their views, parents and carers suggested using a mix of approaches such as home-school communication books, Zoom and telephone. Whilst some staff mentioned challenges in engaging parents and carers, a Family Support Worker (School A) suggested they would be keen to have their voices heard, as they can often feel ignored.

## Discussion

### Developing networks, partnerships and ways of working

In addition to our core stakeholder workshops and scoping research we have taken part in extensive two-way engagement, giving invited talks and discussing the project with the Disabled Children’s Council’s ‘Special Educational Consortium’ (SEC), the School Food All-Party Parliamentary Group, and a webinar for the Nutrition Society Academy. We have also met with the Royal College of Speech and Language Therapists (SLTs), local authority (LA) leads, a school meals consultancy, special school Headteachers, a Head of School Food Curriculum in a large special school, an SLT specialising in dysphagia and complex needs, and an NHS Dental Nurse. Adapt-Ed has contributed to responses (by the Food Education Network and SEC) to the Curriculum and Assessment Review. The Executive Head of a Special Needs Trust of five special schools expressed an interest participating in the future study, 30 EoE schools said in our survey that they were potentially interested in taking part, and we have been contacted by LA leads in areas outside of EoE who want to know more. With additional funding from University of Hertfordshire’s (UH) ESRC Impact Acceleration Account, we are collaborating with public and professional stakeholders to co-produce outputs for different audiences, including a logo competition for children and young people with SEND, an inclusive face to face event and podcast supported by the Herts Young People’s Advisory Group. We are preparing a summary of evidence-informed resources requested by school and LA practitioners in our network and have prepared a Policy Brief that includes recommendations and an invitation to join our future Advisory Board. Crucially, we have strengthened our working relationship with School Food Matters (SFM), who will deliver the intervention and who have identified funding for the future Adapt-Ed HZ intervention.

We have also developed the networks and team required for the future research. We have the support of UH’s Clinical Trials Support Network, who will assist with methodological and statistical expertise in developing the future evaluative study. We have established links with the EoE Regional Research Delivery Network (RDN) that is building a schools network and, with a longer recruiting timeframe that will allow for portfolio adoption, will be able to support recruitment of schools in the future study. We have also added additional members to our research team with subject and methodological expertise in health economics, public health, nutrition and dietetics, speech and language therapy, complex interventions and process evaluation. We are in contact with an advisor at the Specialist Centre for Public Health (University of Leeds) to help support the upcoming funding application.

Importantly, we have grown our understanding of how to work flexibly with children and young people with SEND and their parents and carers in special schools and beyond. We found that working with established mechanisms for eliciting children’s and young people’s voices, such as the school council and SEND Youth Forums, was an effective way to engage with children and young people. Likewise, ‘piggybacking’ on existing activities, such as coffee mornings, for parents/carers was a useful way to reach this group in two schools (A and C). However, as we found in School B, not all schools hold regular sessions such as coffee mornings for parents, or take up may be very low, given large catchments and the long distance from specialist schools that many families live. In this case, parents and carers suggested that offering remote options such as phone calls and Zoom could be helpful. Overall, in terms of methods, we learned that more time would be needed to develop the use of the sunflower metaphor model with children and young people with SEND and that the most effective approach was to work with staff who know pupils best, using a flexible format and toolkit of interactive methods, including Talking Mats, to enable CYP to share their views in ways that work for them ( Skyrme and Woods, 2018; Viper, 2013; Knight et al., 2006; Bloom et al., 2020; O’Connell et al., 2026).

### Collaborative logic model and adaptations to the Nourish intervention

A simple logic model using the metaphor of a sunflower ( Figure 2) was developed collaboratively with our contributors and stakeholders to illustrate how and why the Adapt-Ed HZ intervention may lead to health and wellbeing impacts on children, families and schools. This was based on extensive discussions with our public contributors about their experiences of food in school, as children and young people and as parents and carers of children and young people with SEND. It is also based on extensive discussions in groups and individual meetings and interviews with professional stakeholders working in schools and with schools. For example, children expressed the importance of having a say about food at school and suggested different ways they could do this. Anxiety about food and its immediate impacts on children’s mental health and family relations was raised by parents, carers and school staff as a key issue, and they stressed the importance of strengthening communication with parents to support change. These are some examples of emerging conditions that would need to be met in a future intervention to enhance its chances of success. This logic model is being refined with core stakeholders and, in the next steps of this research programme (feasibility study), it will be implemented in a handful of schools to provide more information to support a full-scale definitive trial.

To underpin the delivery and evaluation of the adapt-ed intervention, a manual is being developed. Some key adaptations to be incorporated in the Adapt-Ed Nourish manual informed by the work for this scoping study are described in Table 12, below. As noted, these include a change of name from Adapt-Ed HZ to Adapt-Ed Nourish:

**Table 12.** Adaptations to tailor Nourish to special school contexts.

What?	How?	Why?
Change of programme name	From ‘Healthy Zones’ to ‘Nourish’	Parents/carers strongly advised to avoid attaching evaluative terms like good/bad and healthy/unhealthy to food
New approach to policy	Schools produce a school food ‘charter’ rather than ‘policy’	Emphasis on individual needs and reasonable adjustments for CYP with EHCPs in special schools. New charter template reflects need for flexibly applied shared ethos not ‘one size fits all’.
Increased programme duration	Extension of duration from 3 to 3.5 terms	School staff and parents/carers emphasise importance of gradual change
Shift in mindset and ways of working	Specific guidance on working in partnership added to manual; extended duration	Nature and variety of children’s needs require POs to ‘work with’ rather than ‘deliver to’ staff and families who are experts and know CYP best.
POs trained in Makaton and flexible methods	Specialist training and mix of consultation methods to support inclusive involvement	Reflecting CYPs capacities, preferred communication methods, and their advice to employ a range of methods to consult children on school food improvement
Tailored ‘menu’ of pilot activities to implement at Phase 2	New activities in manual to address particular contextual barriers faced by CYP with SEND	Scoping reviews and suggestions from CYP, parents/carers and staff informed new activities in flexible components e.g. calming dining strategies, sensory snack times.

### Outline research plan

From the scoping research completed in this ADA, an Adapt-ed Nourish intervention and outline research plan have been developed. These have been co-designed based on our discussions with our Working Group, public co-applicant, public and professional stakeholders and engagement with SFM.

The plan is for a future trial that will adopt a Stepped-Wedge design, as the Adapt-Ed HZ intervention focuses on institutional change. Subject to a feasibility study, it is envisaged that the future trial will consist of three overlapping Work Packages (WP). WP1 will recruit schools to the Stepped-Wedge trial that will implement and evaluate the effectiveness of Adapt-Ed HZ on the health and wellbeing of children with SEND and other outcomes. WP2, a process and outcome evaluation, will use a mix of qualitative and quantitative methods to test whether and how the Adapt-Ed HZ intervention is working. WP3 will develop knowledge mobilisation and impact plans, and public and professional stakeholder involvement activities, which will be woven throughout.

## Conclusions and next steps

Healthy Zones is an intervention for enhancing whole school food environments to promote health and wellbeing that requires implementation and evaluation to determine its effectiveness in special schools. Through our mixed methods scoping research we have learned there is a need and demand for this work. Our secondary analysis of administrative data confirmed that children in special schools are more likely to be entitled to a FSM, but less likely to access one. Our survey and ethnographic research revealed some relevant aspects of special schools’ contexts across different areas of the whole school food environment, what seems to make a difference, the kinds of outcomes and impacts that are seen as desirable and data that seem feasible to collect. Our literature reviews provided us with findings to inform conversations with key stakeholders, to identify instruments and measures which may be appropriate, valid and reliable for the next stage of the project, and to invite members with specialist expertise (nutrition and dietetics, speech and language therapy) to join the team. Through our public involvement with children and young people with SEND and parents and carers, and the input of our public co-applicant, we have developed meaningful relationships and networks, and a lived experience-informed approach; these will underpin plans for flexible and inclusive involvement, and two-way engagement with practice and policy, that will be embedded in the future research from the outset.

Working with children, parents and carers, and professionals in schools and policy roles, this project has provided a wealth of information to inform the refinement of the Healthy Zones approach to suit special schools. It has included extensive public involvement and intensive scoping research to support future research into this topic.

The recommendation of this scoping project is progression towards a definitive trial to evaluate the effectiveness of Adapt-Ed Healthy Zones on the health and well-being of children with SEND, subject to a feasibility study to determine definitive design including outcome measures, data collection and health economic methods.

## Limitations

Our approach to children and young people’s involvement in WP1 was inclusive, but six months was not enough time to establish a bespoke group. However, ongoing engagement activities include those with children and young people aimed at refining the draft logic model and informing next steps. WP3 relied on census day data on FSM take up and achieved a low survey response. However, results were broadly confirmed by ethnographic research and consistent with other evidence (Contact, 2023). Whilst the purpose of WP4 was not to generalise, the small sample size and shorter visit to School C should be noted.

## Future work

Future research is needed to test the Adapt-Ed HZ intervention; subject to the outcome of the feasibility study, we propose that the most appropriate starting point for the design is a Stepped-Wedge trial, as this intervention focuses on institutional change.

## Data Availability

Open Science Framework: Adapt-Ed: co-designing adaptations to a whole school intervention to improve the uptake and impact of food provision in special schools - scoping research for a future trial. https://doi.org/10.17605/OSF.IO/EUHW2 This project contains the following underlying data:
• Parent Coffee Morning Flyer.pdf. (Invitation for parents about taking part in WP4 school coffee mornings). • Parent Consent Form.pdf. (Written consent form for parents taking part in WP4 interviews). • Parent Info Sheet.pdf. (Information sheet for parents about WP4 school visits and taking part in interviews). • Pupil Info Sheet.pdf. (Information sheet for pupils about WP4 school visits and observations). • School Consent Form.pdf. (Written consent form for schools taking part in WP4 school visits). • School Info Sheet.pdf. (Information sheet for schools about taking part in WP4 school visits and observations). • School Visit Social Story.pdf. (Information for schools to help prepare pupils for WP4 school visits). • Survey Info Sheet.pdf. (Information sheet for schools completing the WP3 online school survey). • Survey Invitation.pdf. (Invitation sent to schools about taking part in the WP3 online school survey). • Survey Prize Draw T&C.pdf. (Terms and conditions of the prize draw, as part of the WP3 online school survey). • Survey Questions.pdf. (Questions included in the online school survey, as part of WP3). • School Website Audit.xlsx. (Data from the audit of special schools’ websites across the East of England, as part of WP3). •
SchA_Cook_CookMan.docx. (Anonymised transcript of interview with cook and cook manager in school A, as part of WP4). • SchA_Head.docx. (Anonymised transcript of interview with headteacher in school A, as part of WP4). • SchB_Head.docx. (Anonymised transcript of interview with headteacher in school B, as part of WP4). •
SchC_Heads_Chef.docx. (Anonymised transcript of interview with headteachers and head chef in school C, as part of WP4). Data are available under the terms of the Creative Commons Attribution 4.0 International license (CC-BY 4.0). Due to the exploratory nature of this project, which will be used to inform the design of a subsequent future trial, the anonymised interview transcripts from WP4 (listed above) will be made available via the public repository subject to an embargo, following the publication of the NIHR primary peer review reports and other results. Peer reviewers can obtain access to this data by contacting the corresponding author (Rebecca O’Connell). Due to the potential risk of identifying individual responding schools, and their continued involvement in the next phase of the study (subject to funding and consent to recontact) we did not seek consent to share or archive the underlying data from the online school survey (WP3) beyond the immediate research team. Therefore, the underlying data from the school survey will not be made available for reuse. This is in accordance with our Data Management Plan and ethical approval obtained from the University of Hertfordshire (03562024NovHSET). The underlying data pertaining to the analysis of publicly available administrative datasets (WP3) were obtained from a third-party and are publicly available, as follows:
• Department for Education (2024a). Get Information About Schools (GIAS) [Dataset]. Accessed August 14, 2024. https://get-information-schools.service.gov.uk/
For the purposes of this project, data from GIAS was accessed via the ‘search’ tool using the following steps: Selecting ‘All Establishments’ and unchecking ‘include open schools only’; downloading the ‘full set of data’ for all search results (without filters) in csv format; and later filtering for schools open in August 2024 prior to analysis. The methodology used to analyse the data is presented in the article above. The data search tool can be found here: https://get-information-schools.service.gov.uk/Search?SelectedTab=Establishments
• Department for Education (2024b). Schools, Pupils and their Characteristics (SPC). Academic year 2023/24 [Dataset]. Accessed August 14, 2024. https://explore-education-statistics.service.gov.uk/find-statistics/school-pupils-and-their-characteristics/2023-24
For the purposes of this project, the ‘School level underlying data – 2023/24’ csv data file was used, which can be found via the ‘Additional Supporting Files’ tab on the main SPC 2023–24 webpage (linked above). The methodology used to analyse the data is presented in the article above (see WP3 Methods, (a) combined routinely collected public data).

## Footnotes

^1^
UK education policy is a devolved matter.

^2^
According to the National Audit Office there is ‘no comprehensive, evidence-based explanation’ behind these increases, however it is recognised that autism diagnoses appear to be increasing across developed nations (NAO, 2024:35). Autistic people often experience sensory sensitivities that can impact their relationship with food, leading to selective eating and difficulties with mealtimes.

^3^
Including those involved in delivery in adaptation can help enhance acceptability ( Moore *et al*., 2021, p. 4). ROC also shadowed HZ POs in action in two schools and SFM facilitated a ‘deep dive’ with HZ POs that informed stakeholder discussions and assessment of the evidence.

^4^
A commercial entity that was established within a local authority but operates at ‘arm’s length’ enabling it to trade outside of the constraints of the local authority. See: https://www.localgov.co.uk/The-LATCo-model/51023.

^5^
Scoping review co-authors include public co-applicant AF and a registered dietitian/specialist in dietary assessment who was also an invited member of the project Working Group.

^6^
In a SW-CRT design, stratifying reduces the risk that a simple randomisation will not produce balanced treatment and control groups. However, it is only helpful if we can stratify based on observable characteristics of schools and food provision likely to be strongly correlated with relevant outcomes.

^7^
The East of England includes counties of Essex, Suffolk, Norfolk, Cambridgeshire, and Hertfordshire, and unitary authorities of Bedford, Central Bedfordshire and Luton (historically in Bedfordshire), Southend-on-Sea and Thurrock (historically in Essex) and Peterborough (historically in Cambridgeshire).

^8^
In describing the schools in Table 4 and below we have chosen not to include the county in which each is situated and provide ranges rather than precise figures for some values; this is to protect the identity of schools, staff and pupils.

^9^
As discussed in the paper, and based in part on what we learned during school visits, we are cautious about the validity of these data as an indicator of actual take up, at the level of the individual school.

^10^
The visit was arranged at short notice, shortly before Christmas, due to high staff sickness in a school we’d previously arranged to visit, so we sought to minimise burden on the school.

^11^
To manage and organise the information gathered across the different involvement and engagement workshops and meetings, we drew on the principles of the Framework Approach ( Gale *et al.*, 2013) to construct a simple analysis table. Each row represents a different ‘data point’ (meeting) whilst each column represents a topic or theme. This is mostly a ‘deductive’ process, coding data against predefined topics. However, an ‘other’ column leaves room for inductive insights emerging from each data point.

^12^
This analysis is of the whole of England to preserve statistical power; after controlling for other observable pupil and school characteristics, differences in take up between the East and Rest of England are either statistically insignificant, small at less than a percentage point or both.
