# Supplementary material for: Adapt-Ed: co-designing adaptations to a whole school intervention to improve the uptake and impact of food provision in special schools – scoping research for a future trial
Source: NIHR Open Res. 2026 Aug 1;5:50. Originally published 2025 May 28. [Version 2] doi: 10.3310/nihropenres.13897.2 (PMC13560602; doi:10.3310/nihropenres.13897.2)
Supplement: Peer review history

# Reviewer report: Reviewer response for version 2

**DOI:** 10.3310/nihropenres.15713.r41457

**Published:** 2026-08-24

**Authors:** Riikka Pajulahti

**Affiliations:** 1 University of Helsinki, Helsinki, Finland

The article describes the process and findings of scoping research related to special school settings and the school food environment in these settings to inform a future intervention trial. The process included extensive public and stakeholder involvement, two scoping reviews, exploration of special school context through administrative data, school survey and audit as well as ethnographic case studies. The mixed methods approach with rich data provides understanding of the special school context and the needs and perspectives of the people concerned, thereby giving a good basis for adapting an existing intervention for these settings.

 Overall, the article is well written and clearly structured which helps the reader to understand the extensive process and multiple sources of data. Methods and analyses are described in sufficient detail, and conclusions follow logically the results. The article is an important and valuable addition to the existing literature regarding school meals in different contexts.

 I have some minor suggestions for clarification that are optional for the authors to consider:

**Background:**

- International readers might benefit of a short description of the school food provision system in England, because they may not be familiar with the UK context. This information could include for example, short description of the Universal Infant Free Meals programme, which meals are included in the UIFSM, who are entitled to the means-tested free meals, which meals are included in the means-tested free meals, and whether the system works in the same way in mainstream and special schools etc.

**Methods WP3:**

- the inclusion/exclusion criteria could mention whether there were any restrictions based on the publication year or not
- the statistical software used in the statistical analysis could be briefly mentioned in the methods

**Results **

- **WP2: **last sentence before the results of WP3 (notion of children’s energy intake at school) could have a reference and specification which meals are included in this estimate.
- **WP3 Table 8:** To aid reader’s understanding of the results presented in table 8 (without a need to refer to the text or methods section), perhaps the table notes could explicitly mention which variables were controlled for in columns 1-2. 

**Discussion**

- The name change of the intervention (HZ -> Nourish) is introduced but then used alternately in the following text which caused some confusion.

All data was not available to protect identifiability, but this was appropriately reported and explained.

Is the work clearly and accurately presented and does it cite the current literature?

Yes

If applicable, is the statistical analysis and its interpretation appropriate?

Yes

Are all the source data underlying the results available to ensure full reproducibility?

Partly

Is the study design appropriate and is the work technically sound?

Yes

Are the conclusions drawn adequately supported by the results?

Yes

Are sufficient details of methods and analysis provided to allow replication by others?

Yes

Reviewer Expertise:

Public health nutrition, school meals, interventions, quantitative methods

I confirm that I have read this submission and believe that I have an appropriate level of expertise to confirm that it is of an acceptable scientific standard.

# Reviewer report: Reviewer response for version 2

**DOI:** 10.3310/nihropenres.15713.r41404

**Published:** 2026-08-11

**Authors:** Marie Murphy

**Affiliations:** 1 University of Birmingham, Birmingham, UK

Thank you to the authors for providing additional clarity or detail where suggested. The authors have addressed all comments satisfactorily and I have no further comments. This paper provides a valuable and much needed contribution to the literature on whole school food interventions in special schools.

Is the work clearly and accurately presented and does it cite the current literature?

Yes

If applicable, is the statistical analysis and its interpretation appropriate?

Yes

Are all the source data underlying the results available to ensure full reproducibility?

Partly

Is the study design appropriate and is the work technically sound?

Yes

Are the conclusions drawn adequately supported by the results?

Yes

Are sufficient details of methods and analysis provided to allow replication by others?

Partly

Reviewer Expertise:

Public health nutrition, school food, engaging children and young people with SEND in research

I confirm that I have read this submission and believe that I have an appropriate level of expertise to confirm that it is of an acceptable scientific standard.

# Reviewer report: Reviewer response for version 2

**DOI:** 10.3310/nihropenres.15713.r41403

**Published:** 2026-08-08

**Authors:** Wendy Burton

**Affiliations:** 1 University of York, York, UK

The authors have addressed concerns raised in my peer review report. Research processes are now much clearer and the article is much improved.

Is the work clearly and accurately presented and does it cite the current literature?

Yes

If applicable, is the statistical analysis and its interpretation appropriate?

Not applicable

Are all the source data underlying the results available to ensure full reproducibility?

No

Is the study design appropriate and is the work technically sound?

Yes

Are the conclusions drawn adequately supported by the results?

Partly

Are sufficient details of methods and analysis provided to allow replication by others?

No

Reviewer Expertise:

Qualitative methods, intervention co-design

I confirm that I have read this submission and believe that I have an appropriate level of expertise to confirm that it is of an acceptable scientific standard.

# Reviewer report: Reviewer response for version 1

**DOI:** 10.3310/nihropenres.15104.r39387

**Published:** 2026-03-05

**Authors:** Marie Murphy

**Affiliations:** 1 University of Birmingham, Birmingham, UK

Thank you for the opportunity to review this paper. It provides really valuable insights into both the research context in special schools as well as school food environments and experiences. The reporting of the study is appropriate given the exploratory nature, and the paper is very useful in terms of documenting the process of scoping to inform the future feasibility trail by the study team, as well as other researchers planning future work in this setting. I have a few minor comments / requests for some additional detail:

- WP1 Methods: Sunflower illustration – how did the researchers arrive at this illustration / metaphor? Was this pre-defined or did it come from early discussion with stakeholders?
- Methods WP1 and WP4 – the extent to which the methods supported *meaningful *engagement with CYP with SEND is difficult to assess. Some additional detail would be useful here i.e. in in-person workshops / online groups (WP1) and school council meeting and informal discussions with CYP at case study sites (WP4), what data collection methods were used to gather insights, particularly for children with varying communication needs and physical disabilities? There are some insights in the ethics section (where the authors talk about a range of formats for study information and the use of a visual board and social story for WP4) – however a little more detail on methods for allowing inclusive engagement of CYP would be really valuable to other researchers.
- WP2 results: It would be useful to know the volume of literature and geographical spread for the two scoping reviews – e.g. can the authors report the number of relevant papers identified?
- WP3 results: Reporting of table 9 in the WP3 section is confusing – does this belong in the subsequent section (WP4 results)?
- WP4 results: The authors introduce ADAPT guidance here with no previous introduction. Was this used to shape the design of the scoping research, or the analysis/interpretation? If so, it should be introduced earlier e.g. in background or methods.
- Discussion: Do the research team have any reflections specifically on engaging CYP and parents attending special schools in the future research? This would be a valuable addition to the discussion. This is relevant since in some schools in WP4, engagement was low (School B = 1 parents; School B and C relied on observation and informal child discussion). It would be useful to articulate any learnings taken from this scoping work in relation to increasing child and parent engagement in future work.

Note, not all data are available due to restrictions relating to consent and identifiability - this is reported appropriately by authors where applicable.

Is the work clearly and accurately presented and does it cite the current literature?

Yes

If applicable, is the statistical analysis and its interpretation appropriate?

Yes

Are all the source data underlying the results available to ensure full reproducibility?

Partly

Is the study design appropriate and is the work technically sound?

Yes

Are the conclusions drawn adequately supported by the results?

Yes

Are sufficient details of methods and analysis provided to allow replication by others?

Partly

Reviewer Expertise:

Public health nutrition, school food, engaging children and young people with SEND in research

I confirm that I have read this submission and believe that I have an appropriate level of expertise to confirm that it is of an acceptable scientific standard, however I have significant reservations, as outlined above.

# Response

**Published:** 2026-07-20

**Authors:** Rebecca O'Connell

**Affiliations:** CRIPACC, University of Hertfordshire, Hatfield, England, UK

Sincere thanks for reading the article and providing clear and constructive feedback. We have revised the paper using Track and resubmitted it and set out how we have addressed your comments, below. We hope you will find these satisfactory. 

 Overall, this work provides excellent insight into the existing school food context in special schools and has successfully highlighted what needs to change with adequate conclusions drawn. However, it is not clear what the adapted intervention entails, how it differs from the original intervention and whether / how information gathered in work package 2-4, fed into the co-design of the intervention adaptation, can this be explained? 

- *We have provided a new table (12) that summarises this information and addresses this point. *

WP2 results: It would be useful to know the volume of literature and geographical spread for the two scoping reviews – e.g. can the authors report the number of relevant papers identified? 

- *Thank you for inviting us to provide more detail here. The sunflower was suggested by our collaborators at SFM – appropriate but not food related so less confusing than some other metaphors we reviewed /considered such as baking a cake. We have elaborated on this in the article as follows: ‘In this project, we considered a variety of examples, including ‘baking a cake’; we decided to use a sunflower to present the conditions, steps and outcomes of ‘growing Healthy Zones’, that was suggested by collaborators at charity School Food Matters, as it seemed clear and straightforward to translate into a traditional logic model format ( Figure 1).’ *

Methods WP1 and WP4 – the extent to which the methods supported meaningful engagement with CYP with SEND is difficult to assess. Some additional detail would be useful here i.e. in in-person workshops / online groups (WP1) and school council meeting and informal discussions with CYP at case study sites (WP4), what data collection methods were used to gather insights, particularly for children with varying communication needs and physical disabilities? There are some insights in the ethics section (where the authors talk about a range of formats for study information and the use of a visual board and social story for WP4) – however a little more detail on methods for allowing inclusive engagement of CYP would be really valuable to other researchers. 

- *We have added further detail on methods used to explore the study’s topics with CYP with SEND (page 9). *

WP2 results: It would be useful to know the volume of literature and geographical spread for the two scoping reviews – e.g. can the authors report the number of relevant papers identified? 

- *We have added further details of the search results, including the numbers of articles identified, screened and included. While the scoping reviews are being reported separately in full, we have expanded the summaries presented here to provide greater transparency regarding the evidence base that informed intervention adaptation. *

WP3 results: Reporting of table 9 in the WP3 section is confusing – does this belong in the subsequent section (WP4 results)? 

- *Thank you for spotting this has slipped to the wrong section. We have moved to Wp4 results section.* 

WP4 results: The authors introduce ADAPT guidance here with no previous introduction. Was this used to shape the design of the scoping research, or the analysis/interpretation? If so, it should be introduced earlier e.g. in background or methods. 

- *We introduced the guidance at the start of our description of Wp1 but generically rather than by name, although we did include a reference. We have now replaced  ‘Guidelines’ with ‘The ADAPT guidance’ [on adapting interventions to new contexts recommend collaboration with stakeholders from the earliest stages] ( Moore et al., 2021).] *

Discussion: Do the research team have any reflections specifically on engaging CYP and parents attending special schools in the future research? This would be a valuable addition to the discussion. This is relevant since in some schools in WP4, engagement was low (School B = 1 parents; School B and C relied on observation and informal child discussion). It would be useful to articulate any learnings taken from this scoping work in relation to increasing child and parent engagement in future work. 

- *Thanks for inviting us to elaborate on this. We have changed the first section of the discussion to ‘Developing networks, partnerships and ways of working’ and added a short paragraph at the end of that section summarising what we learned about methodologies and methods for working with CYP and parents/carers in schools. We have also added a short discussion explaining why we did not use the  sunflower logic model with CYP groups to WP1, and reflect on this in the discussion. *

# Reviewer report: Reviewer response for version 1

**DOI:** 10.3310/nihropenres.15104.r36043

**Published:** 2025-11-03

**Authors:** Wendy Burton

**Affiliations:** 1 University of York, York, UK

This article details scoping research that was undertaken as part of a NIHR Application Development Award. The work aims to understand school food provision for children with SEND and how the whole school food environment can be improved. The article describes four work packages, 1) a stakeholder involvement workstream to support understanding of how an existing whole school food approach (Healthy Zones) can be adapted / improved, 2) two scoping reviews to identify existing food interventions for children and young people with SEND along with appropriate impact assessment tools, 3) analysis of a publicly available data and an online survey to explore the relationship between school characteristics and uptake of free school meals, and 4) an ethnographic case study of three special schools.

 In work package one, a logic model was co-produced which sets out how to make nutritious food accessible throughout the day to every school pupil. The scoping reviews identified potential interventions to improve healthy eating, feeding and diets of children and young people with SEND along with potential assessment tools to be used in an eventual evaluation of the adapted intervention. Work package 3 identified that uptake of free school meals in special schools lags behind other schools, and proposed factors that influence uptake (including meal cost and time available for lunch). The case study research described similarities and differences between schools in respect of their whole school food environment, including educational philosophy and approach and policies and leadership.

 This background is well written and provides a clear rationale for the programme of work as well as setting out the aims and objectives of the overall project.

 The methods section is well written and describes the large volume of work in a manageable format. However, some details are missing that would be useful for the reader:

- Work undertaken within work package one is described as the ‘co-design of adaptations to the Healthy Zones approach’, but details and examples of the existing Healthy Zones approach are not provided to justify why it cannot be implemented in special schools in its current form.
- The scoping review methods are lacking important details which prevent the work from being replicated, such as the search strategies and inclusion and exclusion criteria.
- Work package 4 describes an ‘ethnographic case study’, but the methods described do not appear consistent with this methodology, for example researchers do not appear to have spent a sufficient amount of time in the field to become immersed in the setting. Can the rationale for the approach taken be justified along with some explanation of how the approach differs from a traditional ethnographic study? 

The results are clear and well presented, but the following details need to be clarified or added in regard to work package 1 and 2:

- In work package one, the process used to co-design the logic model is well detailed, including the summary of discussions held within co-design meetings. However, it is not clear what the adapted version of the Healthy Zones intervention would entail, e.g., what will be delivered, when and by whom and how does this differ from the original intervention. Can this information be provided or summarised, perhaps using the TIDieR checklist?
- The scoping review results are not adequately reported, for example, how many studies were identified and deemed eligible / ineligible, what were the characteristics of the studies? Can the findings of the reviews be provided as additional files using a reporting standard (such as PRISMA extension for scoping reviews (PRISMA-ScR)?

Work package 3 includes analysis of routine data (with a large sample) and a regression analysis to consider factors that may influence uptake. The survey data is also descriptive.  The sample size is relatively small, but the authors have accounted for this to some degree, but comparing characteristics to non-responding schools, and by highlighting this caveat in the text.

 The results of the case study ethnography are well presented in Table 9 to provide an overview of the school contexts in respect to the Whole School Food Environment Framework. The accompanying text is also well written and provides a good account of the findings.

 Overall, this work provides excellent insight into the existing school food context in special schools and has successfully highlighted what needs to change with adequate conclusions drawn. However, it is not clear what the adapted intervention entails, how it differs from the original intervention and whether / how information gathered in work package 2-4, fed into the co-design of the intervention adaptation, can this be explained?

 Plans for future work are well articulated and justified.

Is the work clearly and accurately presented and does it cite the current literature?

Yes

If applicable, is the statistical analysis and its interpretation appropriate?

Not applicable

Are all the source data underlying the results available to ensure full reproducibility?

No

Is the study design appropriate and is the work technically sound?

Yes

Are the conclusions drawn adequately supported by the results?

Partly

Are sufficient details of methods and analysis provided to allow replication by others?

No

Reviewer Expertise:

Qualitative methods, intervention co-design

I confirm that I have read this submission and believe that I have an appropriate level of expertise to confirm that it is of an acceptable scientific standard, however I have significant reservations, as outlined above.

# Response

**Published:** 2026-07-20

**Authors:** Rebecca O'Connell

**Affiliations:** CRIPACC, University of Hertfordshire, Hatfield, England, UK

Sincere thanks for reading the article and providing clear and constructive feedback. We have revised the paper using Track and resubmitted it and set out how we have addressed your comments, below. We hope you will find these satisfactory.  

 Work undertaken within work package one is described as the ‘co-design of adaptations to the Healthy Zones approach’, but details and examples of the existing Healthy Zones approach are not provided to justify why it cannot be implemented in special schools in its current form  

- *We explored through the work what were relevant aspects of special school contexts, and what changes might be needed as a result. We have now added a table (Table 12) showing key changes, and how these are underpinned by what we learned, that we hope addresses this point. *

The scoping review methods are lacking important details which prevent the work from being replicated, such as the search strategies and inclusion and exclusion criteria. 

- *We have now included an inclusion and exclusion table using the PCC (Participants/ Concept/ Context) mnemonic for scoping reviews 1 and 2. We have also added further details of the search results, including the numbers of articles identified, screened and included. While the scoping reviews are being reported separately in full, we have expanded the summaries presented here to provide greater transparency regarding the evidence base that informed intervention adaptation. *

Work package 4 describes an ‘ethnographic case study’, but the methods described do not appear consistent with this methodology, for example researchers do not appear to have spent a sufficient amount of time in the field to become immersed in the setting. Can the rationale for the approach taken be justified along with some explanation of how the approach differs from a traditional ethnographic study? 

- *This is an important clarification. We have amended, adding some finance and a reference as follows: ‘Our approach, using a mix of qualitative methods including participant observation, is consistent with a broadly ethnographic design, however we did not have the resources in this ADA to fully immerse ourselves in school settings over an extended period of time. Rather our approach was more akin to the early stages of a larger ethnographic study, sometimes referred to as ‘scoping the field’. We made short visits to schools, purposively sampling days, situations and people, to familiarise ourselves with the settings and identify relevant aspects of context to inform adaptations to the intervention and the future study (O’Connell, 2016)’. *

In work package one, the process used to co-design the logic model is well detailed, including the summary of discussions held within co-design meetings. However, it is not clear what the adapted version of the Healthy Zones intervention would entail, e.g., what will be delivered, when and by whom and how does this differ from the original intervention. Can this information be provided or summarised, perhaps using the TIDieR checklist? 

- *We have added a new table (12) to address this point. *

The scoping review results are not adequately reported, for example, how many studies were identified and deemed eligible / ineligible, what were the characteristics of the studies? Can the findings of the reviews be provided as additional files using a reporting standard (such as PRISMA extension for scoping reviews (PRISMA-ScR)? 

- *We have added further details of the search results, including the numbers of articles identified, screened and included. While the scoping reviews are being reported separately in full, we have expanded the summaries presented here to provide greater transparency regarding the evidence base that informed intervention adaptation. *
